# Defining human-AI teaming the human-centered way: a scoping review and network analysis

**DOI:** 10.3389/frai.2023.1250725

**Published:** 2023-09-29

**Authors:** Sophie Berretta, Alina Tausch, Greta Ontrup, Björn Gilles, Corinna Peifer, Annette Kluge

**Affiliations:** ^1^Department of Psychology, Organizational, and Business Psychology, Ruhr University Bochum, Bochum, Germany; ^2^Department of Psychology I, University of Lübeck, Lübeck, Germany

**Keywords:** artificial intelligence, human-centered AI, network analysis, bibliometric analysis, bibliometric coupling, work psychology, human-AI teaming, humane work

## Abstract

**Introduction:**

With the advancement of technology and the increasing utilization of AI, the nature of human work is evolving, requiring individuals to collaborate not only with other humans but also with AI technologies to accomplish complex goals. This requires a shift in perspective from technology-driven questions to a human-centered research and design agenda putting people and evolving teams in the center of attention. A socio-technical approach is needed to view AI as more than just a technological tool, but as a team member, leading to the emergence of human-AI teaming (HAIT). In this new form of work, humans and AI synergistically combine their respective capabilities to accomplish shared goals.

**Methods:**

The aim of our work is to uncover current research streams on HAIT and derive a unified understanding of the construct through a bibliometric network analysis, a scoping review and synthetization of a definition from a socio-technical point of view. In addition, antecedents and outcomes examined in the literature are extracted to guide future research in this field.

**Results:**

Through network analysis, five clusters with different research focuses on HAIT were identified. These clusters revolve around (1) human and (2) task-dependent variables, (3) AI explainability, (4) AI-driven robotic systems, and (5) the effects of AI performance on human perception. Despite these diverse research focuses, the current body of literature is predominantly driven by a technology-centric and engineering perspective, with no consistent definition or terminology of HAIT emerging to date.

**Discussion:**

We propose a unifying definition combining a human-centered and team-oriented perspective as well as summarize what is still needed in future research regarding HAIT. Thus, this work contributes to support the idea of the Frontiers Research Topic of a theoretical and conceptual basis for human work with AI systems.

## 1. Introduction

With the uprise of technologies based on artificial intelligence (AI) in everyday professional life (McNeese et al., 2021), human work is increasingly affected by the use of AI, with the growing need to cooperate or even team up with it. AI technologies describe intelligent systems executing human cognitive functions such as learning, interacting, solving problems, and making decisions, which is an enabler for using them in a similarly flexible manner as human employees (e.g., Huang et al., 2019; Dellermann et al., 2021). Thus, the emerging capabilities of AI technologies allow them to be implemented directly in team processes with other artificial and human agents or to overtake functions that support humans in a way team partners would. Such can be referred to as human-AI teaming (HAIT; McNeese et al., 2018). HAIT constitutes a human-centered approach to AI implementation at work, as its aspiration is to leverage the respective strengths of each party. The diverse but complementary capabilities of human-AI teams foster effective collaboration and enable the achievement of complex goals while ensuring human wellbeing, motivation, and productivity (Kluge et al., 2021). Other synergies resulting from human-AI teaming facilitate strategic decision making (Aversa et al., 2018), the development of individual capabilities, and thus employee motivation in the long term (Hughes et al., 2019).

Up to now, the concept of HAIT has been investigated from various disciplinary perspectives, e.g., engineering, data sciences or psychology (Wilkens et al., 2021). An integration of these perspectives seems necessary at this point to design complex work systems as human-AI teams with technical, human, task, organizational, process-related, and ethical factors in mind (Kusters et al., 2020). In addition to this, a conceptual approach with a unifying definition is needed to unite research happening under different terms, but with a potentially similar concept behind it. To evolve from multi- to interdisciplinarity, the field of HAIT research needs to overcome several obstacles:

(1) The discipline-specific definitions and understandings of HAIT have to be brought together or separated clearly.
(2) Different terms used for the same concept, e.g., human-autonomy teaming (O'Neill et al., 2022) and human-AI collaboration (Vössing et al., 2022), have to be identified to enable knowledge transfer and integration of empirical and theoretical work.
(3) The perspectives on either the technology or the human should be seen as complementary, not as opposing.

As “construct confusion can [...] create difficulty in building a cohesive body of scientific literature” (O'Neill et al., 2022, p. 905), it is essential that different disciplines find the same language to talk about the challenges of designing, implementing and using AI as a teammate at work. Therefore, the goal of this scoping review is to examine the extent, range, and nature of current research activities on HAIT. Specifically, we want to give an overview of the definitory understandings of HAIT and of the current state of empirically investigated and theoretically discussed antecedents and outcomes within the different disciplines. Based on a bibliometric network analysis, research communities will be mapped and analyzed regarding their similarities and differences in the understanding of HAIT and related research activities. By this, our scoping review reaches synergistic insights and identifies research gaps in examining human-AI teams, promoting the formation of a common understanding.

## 2. Theoretical background: human-AI teaming in the workplace

As technologies progress and AI becomes more widely applied, humans will no longer work together only with other humans but will increasingly need to use, interact with and leverage AI technologies to achieve complex goals. Increasingly “smart” AI technologies entail characteristics that require new forms of work and cooperation between human and technology (Wang et al., 2021), developing from “just” technological tools to teammates to human workers (Seeber et al., 2020). According to the CASA-paradigm, people tend to perceive *c*omputers *a*s *s*ocial *a*ctors (Nass et al., 1996), which is probably even more true with highly autonomous technologies driven by AI, being seen as very agentic. This opens opportunities to move the understanding of AI as a helpful technological application to a team member that interdependently works with the employee toward a shared and valued goal (Rix, 2022). Thus, human-AI teams evolve as a new form of work, pairing human workforce and abilities with that of AI.

Why is a shift in parameters needed? Our proposed answer is that that it offers a new, humane attempt toward AI implementation at work that respects employees' needs, feeling of belongingness and experience (Kluge et al., 2021). Additionally, employees' acceptance, and a positive attitude in working with an AI can improve when it is seen as a teammate (see, e.g., Walliser et al., 2019). Thus, HAIT provides an opportunity to create attractive and sustainable workplaces by harnessing people's capabilities and enabling learning and mutual support. This in turn leads to synergies (Kluge et al., 2021), increased motivation and wellbeing on the part of humans, by spending more time on identity-forming and creative tasks, while safety-critical and monotonous tasks can be handed over to the technology (Jarrahi, 2018; Kluge et al., 2021; Berretta et al., 2023). In addition to the possibility of creating human-centered workplaces, the expected increase in efficiency and performance due to complementary capabilities of humans and AI technologies, described as synergies, are further important reasons for the parameter shift (Dubey et al., 2020; Kluge et al., 2021).

However, those advantages connected to the human workforce and the performance do not just come naturally when pairing humans with AI systems. The National Academies of Sciences, Engineering, and Medicine (2021) defines four conditions for a human-AI team to profit from these synergies:

(1) The human part has to be able to understand and anticipate the behaviors of the deployed intelligent agents.
(2) To ensure appropriate use of AI systems, the human should be able to establish an appropriate relationship of trust.
(3) The human part can make accurate decisions when using the output information of the deployed systems and
(4) has the ability to control and handle the systems appropriately.

These conditions demonstrate that successful teaming depends on technical (e.g., design of the AI system) as well as human-related dimensions (e.g., trust in the system) and additionally requires interaction/teamwork issues (e.g., form of collaboration). This makes HAIT an inherently multidisciplinary field, that should be explored in the spirit of joint optimization to achieve positive results in all dimensions (Vecchio and Appelbaum, 1995). Nevertheless, joint consideration and optimization is still not common practice in the development of technologies or the design of work systems (Parker et al., 2017), so that much research looks at HAIT solely from one perspective. The following section introduces two perspectives on teams in work contexts relevant for the proposed, joint HAIT approach.

### 2.1. Human-technology teaming

The field of human-technology teaming encompasses a number of established concepts, including human-machine interaction (e.g., Navarro et al., 2018) or human-automation interaction (e.g., Parasuraman et al., 2000). These constructs can, but do not have to, include aspects of teaming: they describe a meta-level of people working in some kind of contact with technologies. Concepts further specify on two different aspects: the interaction aspect and the technology aspect. The term “interaction” as a broad concept is increasingly replaced by terms trying to detail the type of interaction such as co-existence, cooperation and collaboration (Schmidtler et al., 2015), usually understood as increasingly close and interdependent contact. Maximally interdependent collaboration including an additional aspect of social bonding (team or group cohesion, see Casey-Campbell and Martens, 2009) is called teaming. In terms of the technology aspect, a range of categories exists from general terms like technology, machines or automation, which can be broad or specific, depending on the context (Lee and See, 2004). More specific categories include autonomy, referring to adaptive, self-governed learning technologies (Lyons et al., 2021), robots or AI.

A recent and central concept in this research field is human-autonomy teaming, as introduced by O'Neill et al. (2022) in their review. Although using a different term than HAIT, this concept plays a crucial role in consolidating and unifying research on the teaming of humans and autonomous, AI-driven systems. Their defining elements of human-autonomy teaming include:

(1) a machine with high agency,
(2) communicativeness of the autonomy,
(3) conveying information about its intent,
(4) evolving shared mental models,
(5) and interdependence between humans and the machines (O'Neill et al., 2022).

However, there are several critical aspects to consider in this review: The term “human-autonomy teaming” can elicit associations that may not contribute to the construct of HAIT. The definition of autonomy varies between different fields and the term alone can be misleading, as it can be understood as the human's autonomy, the autonomy of a technical agent, or as the degree of autonomy in the relationship. Additionally, O'Neill et al.'s (2022) reliance on the levels of automation concept (Parasuraman et al., 2000) reveals a blind spot in human-centeredness, because the theory fails to consider different perspectives (Navarro et al., 2018) and is not selective enough to describe complex human-machine interactions. Furthermore, the review primarily focuses on empirical research, neglecting conceptual work on teaming between humans and autonomous agents. As a result, the idea of teaming is—despite the name—not as prominent as expected, and the dynamic, mutually supportive aspect of teams is overshadowed by the emphasis on technological capabilities for human-autonomy teaming.

In addition to the emerging problem of research focusing solely on technology aspects, which is important, but insufficient to fully describe and understand a multidimensional system like HAIT, different definitions exist to describe what we understand by human-AI teams. Besides the already mentioned definition of human-autonomy teaming, Cuevas et al. (2007) for example describe HAIT as “one or more people and one or more AI systems requiring collaboration and coordination to achieve successful task completion” (p. 64). Demir et al. (2021, p. 696) define that in HAIT “human and autonomous teammates promptly interact with one another in response to information flow from one team member to another, adapt to the dynamic task, and achieve common goals”. While these definitions share elements, such as the idea of working toward a common goal with human and autonomous agents, there are also dissimilarities among the definitions, for example, in the terminology used, as seemingly similar terms like interaction and collaboration represent different constructs (Wang et al., 2021).

In an evolving research field, terminology ambiguity can inspire different research foci, but also pose challenges. Different emerging research fields might refer to the same phenomenon using various terms (i.e., human-AI-teaming vs. human-autonomy-teaming or interaction vs. teaming), which is known as jangle-fallacy and can cause problems in research (Flake and Fried, 2020). Such conceptual blurring may hinder interdisciplinary exchange and the integration of findings from different disciplines due to divergent terminology (O'Neill et al., 2022).

### 2.2. Human-human teaming

Another important perspective to consider is that of human teams, which forms the foundation of team research. Due to its roots in psychology and social sciences, the perspective on teams is traditionally a human-centered one, implying relevant insights on the blind spot of human-technology teaming research. The term “team” refers to two or more individuals interacting independently to reach a common goal and experiencing a sense of “us” (Kauffeld, 2001). Each team member is assigned a specific role or function, usually for a limited lifespan (Salas et al., 2000). Teamwork allows for the combination of knowledge, skills, and specializations, the sharing of larger tasks, mutual support in problem-solving or task execution, and the development of social structures (Kozlowski and Bell, 2012).

The roots of research on human teams can be traced to the Hawthorne studies conducted in the 1920s and 1930s (Mathieu et al., 2017). Originally designed to examine the influence of physical work conditions (Roethlisberger and Dickson, 1939), these studies unexpectedly revealed the impact of group dynamics on performance outcomes, leading to a shift in focus toward interpersonal relationships between workers and managers (Sundstrom et al., 2000). In this way, psychology's understanding of teamwork and its effects has since stimulated extensive theory and research on group phenomena in the workplace (Mathieu et al., 2017). Following over a century of research, human teamwork, once a “black box” (Salas et al., 2000, p. 341), is now well-defined and understood. According to Salas et al. (2000), teams are characterized by three main elements: Firstly, team members have to be able to coordinate and adapt to each other's requirements in order to work effectively as a team. Secondly, communication between team members is crucial, particular in uncertain and dynamic environments, where information exchange is vital. Lastly, a shared mental model is essential for teamwork, enabling team members to align their efforts toward a common goal and motivate each other. Moreover, successful teamwork requires specific skills, such as adaptability, shared situational awareness, team management, communication, decision-making, coordination, feedback, and interpersonal skills (Cannon-Bowers et al., 1995, see Supplementary Table 1 for concept definitions).

Commonalities of human-human teams and human-AI teams have already been identified in terms of relevant features and characteristics that contribute to satisfactory performance, including shared mental models, team cognitions, situational awareness and communication (Demir et al., 2021). Using human-human teams research insights as a basis for HAIT offers access to well-established and tested theories and definitions, but leaves unclarities in the questions which characteristics and findings can be effectively transferred to HAIT research and what the vital existing differences are (McNeese et al., 2021).

### 2.3. Combining human-technology and human-human teaming in a human-centered way

A consideration of both the human-human and human-technology teaming perspectives serves as a useful and necessary starting point for exploring human-AI teams. In order to advance our understanding, it is crucial to combine the findings from these perspectives and integrate them within a socio-technical systems approach. The concept of socio-technical systems recognizes that the human part is intricately linked to the technological elements in the workplace, with both systems influencing and conditioning each other (Emery, 1993). Therefore, a comprehensive understanding of human-AI teams can only be achieved through an integrative perspective that considers the interplay between humans and technology, as well as previous insights from both domains regarding teaming. In our review, we aim to address the lack of integration by…

- establishing the term human-AI teaming (HAIT) as an umbrella term for teamwork with any sort of artificially intelligent (partially), autonomously acting system.
- omitting a theoretical basement for embedding our literature search and analysis. We want to neutrally identify how (different) communities understand and use HAIT and what might be the core to it, without pre-assumptions on the characteristics.
- taking a human-centered perspective and using the ideas of socio-technical system designs to discuss our findings, anyways.
- including a broad range of scientific literature, which contains conceptual and theoretical papers—thereby being able to cover a deeper examination of HAIT-related constructs.
- seeing if the understanding of teaming has developed since the review by O'Neill et al. (2022) and if there are papers considering especially the team level and dynamics associated with agents sharing tasks.

### 2.4. Rational for this study: research questions and intentions

The goal of this paper is to examine the scope, breadth, and nature of the most current research on HAIT. In this context, we are interested in understanding the emerging research field, the streams and disciplines involved, by visualizing and analyzing current research streams using clusters based on a bibliometric network analysis (“who cites who”). The aim is to use mathematical methods to capture and analyze the relationships between pieces of literature, thereby representing the quantity of original research and its citation dependencies to related publications (Kho and Brouwers, 2012). The investigation of resulting networks can reveal research streams and trends in terms of content and methodology (Donthu et al., 2020). Concisely, the objective of the network analysis is to investigate the following research question:

*RQ1: Which clusters can be differentiated regarding interdisciplinary and current human-AI teaming research based on their relation in the bibliometric citation network?*

Further, the publications of the identified clusters will be examined based on a scoping review concerning the definitory understanding of human-AI teams as well as their empirically investigated or theoretically discussed antecedents and outcomes. This should contribute to answering the subsequent research questions:

*RQ2: Which understandings of human-AI teaming emerge from each cluster in the network?*

*RQ3: Which antecedents and outcomes of human-AI teaming are currently empirically investigated or theoretically discussed?*

This second part of the analysis should lead to a consideration of the quality of publications in the network in addition to the quantity within the network analysis (Kho and Brouwers, 2012). We want to give an overview of what is seen as the current core of HAIT within different research streams and identify differences and commonalities. On the one hand, making differences in the understanding of HAIT explicit is important, as it allows future research to develop into decidedly distinct research strands. On the other hand, the identification of similarities creates a basis for the development of a common language about HAIT, which will allow the establishment of common ground in the future so that the interdisciplinary exchange on what HAIT is and can be grows in stringency. To also contribute to this aspect, we aim to identify a definition of HAIT that serves the need for a common ground. In doing so, the definition is intended to extend that of O'Neill et al. (2022), reflecting the latest state of closely related research as well as addressing and considering the problems identified earlier. If we are not able to find this kind of a definition within the literature that focuses on the teaming aspect, we want to use the insights from our research to newly develop such a definition of HAIT. Thus, our fourth research question, which we will be able to answer after collecting all other results and discussing their implications, is:

*RQ4: How can we define HAIT in a way that is able to bridge different research streams?*

This is expected to help researchers from different disciplines finding a shared ground in definitions and concepts and explicating divergences in understanding. By identifying the current state of research streams and corresponding understandings of HAIT, as well as the antecedents and outcomes, synergistic insights and research gaps can be identified. A unifying definition will further help stimulate and align further research on this topic.

## 3. Materials and methods

To identify research networks and to analyze their findings on HAIT, the methods of bibliometric network analysis and scoping review were combined. The pre-registration for this study can be accessed here: https://doi.org/10.23668/psycharchives.12496.

### 3.1. Literature search

**Figure 1:**
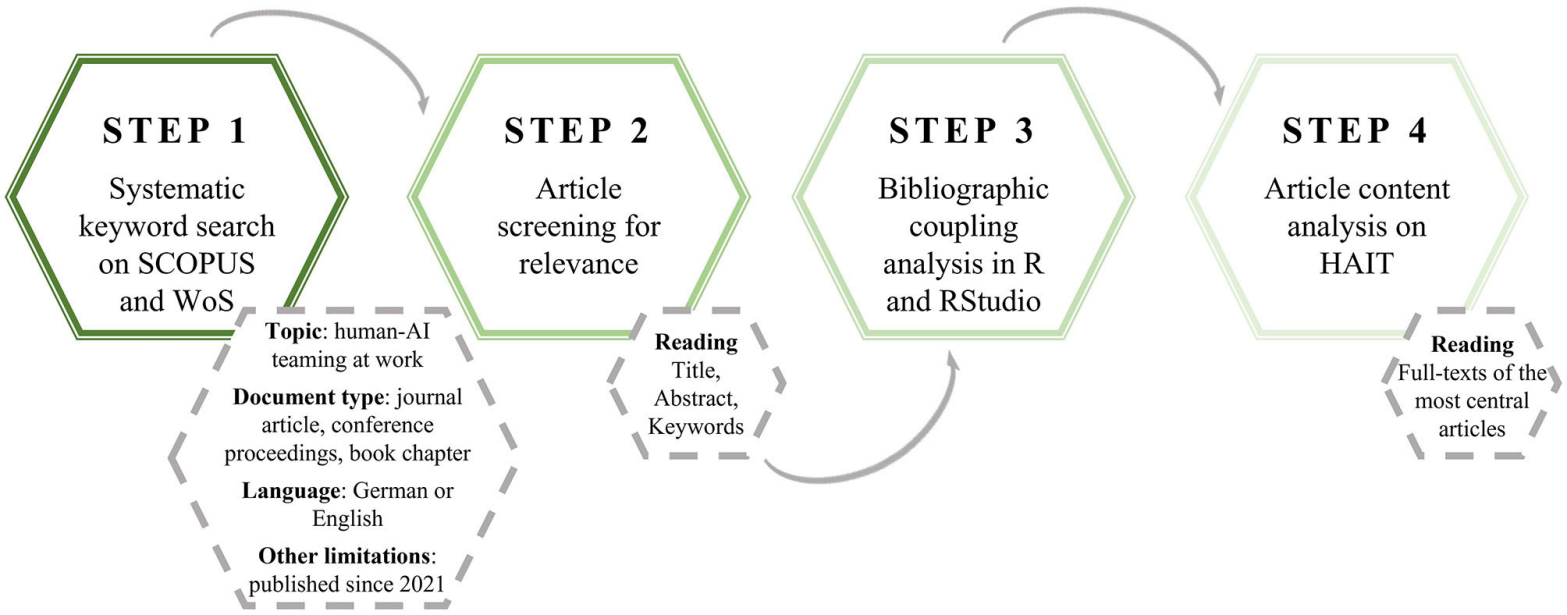
Illustration of the procedure. WoS, web of science; HAIT, human-AI teaming.

The basis for the network analysis and the scoping review was a literature search in Clarivate Analytic's Web of Science (WoS) and Elsevier's Scopus (Scopus) databases. Those were chosen because they represent the main databases for general-purpose scientific publications, spanning articles, conference proceedings and more (Kumpulainen and Seppänen, 2022). The process of the literature search was conducted and is reported according to the PRISMA reporting Guidelines for systematic reviews (Moher et al., 2009), more specifically the extension for scoping reviews (PRISMA-ScR; Tricco et al., 2018). Figure 1 provides an overview of the integrated procedure.

**Figure 2:**
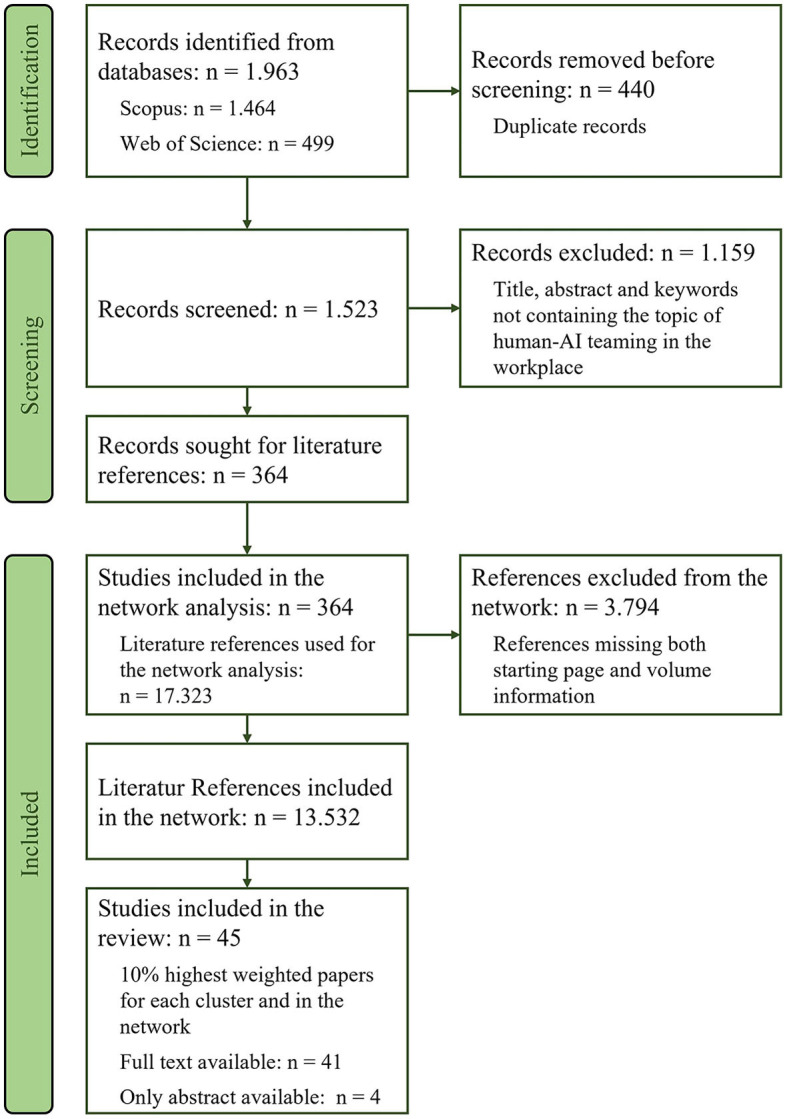
PRISMA-diagram of the conducted literature screening process.

The literature search was conducted on the 25.01.2023. The keywords for our literature search (see Table 1) were chosen to include all literature in the databases that relates to HAIT in the workplace. Thus, the components “human,” “AI,” “teamwork,” and “work” all needed to be present in any (synonymous) form. Furthermore, only articles published since the year 2021 were extracted. This limited time frame was chosen as the goal was to map the most current research front, using the European industrial strategy “Industry 5.0” (Breque et al., 2021) as s starting point. Its focus on humans, their needs, and capabilities instead of technological system specifications represents a shift in attention to the individual that is accompanied by the explicit mention of creating a team of human(s) and technical system(s) (Breque et al., 2021), therefore marking a good starting point of a joint human-AI teaming understanding. Accordingly, only the most current literature published since the introduction of Industry 5.0 and not yet included in the review of O'Neill and colleagues is taken into account in our review (note that by analyzing the references in bibliometric coupling and qualitatively evaluating the referred concepts of HAIT, we also gain information on older important literature). Included text types were peer-reviewed journal articles, conference proceedings and book chapters (not limited to empirical articles) in English or German language. As shown in the PRISMA-diagram in Figure 2, the search resulted in *n* = 1,963 articles being retrieved. After removing *n* = 440 duplicates, abstract-screening was conducted using the web-tool Rayyan (Ouzzani et al., 2016). In case of duplicates, the WoS version was kept for its preferable data structure.

**Table 1:** Used search-terms for the database-search.

**Human**	**AI**	**Teaming**	**Work**
“human”	“AI”	“team^*^”	“work”
“people”	“artificial intelligence”	“collaborat^*^”	“occupation”
“employee”	“machine learning”	“cooperation”	“profession”
“Mensch”	“synthetic agent”	“symbiosis”	“job”
“Mitarbeiter^*^”	“autonomous agent”	“alliance”	“Arbeit”
“Beschäftigte^*^”	“KI”	“coalition”	“Beruf^*^”
	“Künstliche Intelligenz”	“partner^*^”	
	“maschinelles lernen”	“Kollaboration”	
		“Kooperation”	
		“Symbiose”	
		“Tandem”	

Four categories of terms were used: Human, AI, Teaming and Work. Terms inside a category were connected by the search operator “OR” and Categories themselves were connected by the “AND” operator. The asterisk serves as a wildcard for different endings to a common word stem.

Six researchers familiar with the subject screened the abstracts, with every article being judged by at least two blind raters. Articles not dealing with the topic “human-AI teaming in the workplace” or being incorrectly labeled in the database and not fitting our eligibility criteria were excluded. In case of disagreement or uncertainty, raters discussed and compared their reasoning and decided on a shared decision, and/or consulted the other raters. In total, 1,159 articles (76%) were marked for exclusion. Exclusion criteria were: (a) publication in another language than English or German, (b) publication form of a book (monography or anthology), (c) work published before 2021, (d) work not addressing human-AI-teaming in title, abstract or keywords, (e) work not addressing work context in title, abstract or keywords. The remaining articles (*n* = 364, 24%) were used in the network analysis (see Figure 2).

### 3.2. Bibliometric mapping approach and clustering algorithm

To map and cluster the included literature and thus describe the network that structures the research field of HAIT, a bibliometric mapping approach and clustering algorithm had to be chosen. Networks consist of publications that are mapped, called *nodes*, and the connection between those nodes, which are called *edges* (Hevey, 2018). Which publications appear in the nodes and how the edges are formed depends on the mapping approach used. The variety includes direct citation, bibliometric coupling, and co-citation networks (Boyack and Klavans, 2010), but bibliometric coupling analysis has been shown to be the most accurate (Boyack and Klavans, 2010). It works by first choosing a sample of papers, serving as the network nodes. The edges are then created by comparing the references of the node-papers, adding edges between two publications if they share references (Jarneving, 2005). Thus, the newest publications are mapped, while the cited older publications themselves are not included in the network (Boyack and Klavans, 2010; Donthu et al., 2021). Since our goal was to map and cluster the current research front, we chose bibliometric coupling for our network analysis approach.

The article metadata from WoS and Scopus were prepared for network analysis using R (version 4.2, R Core Team, 2022), as well as their reference lists. We did this in a way that the first author, including initials, the publishing year, the starting page, and the volume were extracted from all cited references. This information was then combined in a new format string. In total, *n* = 17.323 references containing at least first author and year were generated. Of those, 8.955 references contained missing data about the starting page, the volume or both. To minimize the risk of two different articles randomly having the same reference string, we excluded all references that missed both volume and starting page information (*n* = 3,794). We kept all references that only had either starting page (*n* = 1,384) or volume (*n* = 3,794) information missing, due to a low probability and influence of single duplicates.

### 3.3. Network analysis

Using the newly created format, we conducted a coupling network analysis using R and the packages *igraph* (version 1.3.1; Aria and Cuccurullo, 2017) and *bibliometrix* (version 4.1.0; Csárdi et al., 2023). The used code can be accessed here: https://github.com/BjoernGilles/HAIT-Network-Analysis. Bibliometrix was used to create the first weighted network with no normalization. Then it was converted into igraph format, removing any isolated edges with degree = 0. The degree centrality refers to the number of edges a node is connected by to other nodes, while the weighted degree centrality adapts this measure by multiplying it with the strength of the edge (Donthu et al., 2021). Then, the multilevel community clustering algorithm was used to identify the dominant clusters. Multilevel-clustering was chosen since the network's mixing parameter was impossible to predict a-priori and since it shows stable performance for a large range of clustering structures (Yang et al., 2016). The stability of our clustering solution was checked by comparing our results with 10,000 recalculations of the multilevel-algorithm on our network-data.

Afterwards, all clusters containing ≥ 20 nodes were selected and split into subgraphs. The top 10% of papers with the highest weighted degree of each subgraph were selected for qualitative content analysis (representing the most connected papers for each cluster). Additionally, we selected the 10% papers with the highest weighted degree in the main graph for content analysis (i.e., representing the most connected papers over all the clusters, i.e., in the whole network). We decided to use the weighted degree as a measure for centrality, because our goal was to identify the most representative and strongest connected nodes in each cluster.

### 3.4. Content analysis

To analyze the content of our literature network and the respective clusters, we chose the *scoping review* approach. It is defined as a systematic process to map existing literature on a research object with the distinctiveness of including all kinds of literature with relevance to the topic, not only empirical work (Arksey and O'Malley, 2005). It is especially of use with emerging topics and evolving research questions (Armstrong et al., 2011) and to identify or describe certain concepts (Munn et al., 2018). Its aims are to show which evidence is present, clarify concepts, how they are defined and what their characteristics are, explore research methods and find knowledge (Munn et al., 2018)—and thus, match our research goals. Whilst this approach lead our systematic literature selection, as described before, it also was our guideline in analyzing the content of the network and the selected publications within.

To understand the network that the respective analysis produced, we looked at the 10% publications with the highest weighted degree in each cluster, analyzing both the metadata such as authors and journals involved, and the content of those papers. For this, we read the full texts of all those publications that were available to us (*n* = 41), as well as the abstracts of the literature without full-text access (*n* = 4). To find the literature's full texts, we looked into the databases and journals that were available to us as university members as well as for open access publication websites, e.g., on Research Gate. For those articles we could not find initially, we contacted the authors. Nevertheless, we could still not get access to four papers, namely Jiang et al. (2022) (cluster 1), Silva et al. (2022) (cluster 3), Tsai et al. (2022) (central within network), Zhang and Amos (2023) (central within network). For those, as they were amongst the most connected publications based on the bibliographic clustering, we considered at least information from the title and abstract.

We first synthesized the main topics of each of the clusters, identifying a common sense or connecting elements within. To then differentiate the clusters, we described them based on standardized categories including the perspective of the articles, research methods used, forms of AI described, role and understanding of AI, terms for and understandings of HAIT and contexts under examination. This, in addition to the network analysis itself, helped to answer RQ1 on clusters within interdisciplinary HAIT research.

The focus then was on answering RQ2 about the understandings of HAIT represented within the network. For this, we read the full texts central within the clusters and within the whole network, marking all phrases describing, defining, or giving terms for HAIT, presenting the results on a descriptive base. We as well-sorted the network-related papers by the terms they used and the degree of conceptuality behind the constructs to get an idea of terminology across the network.

To answer RQ3 about antecedents and outcomes connected to HAIT, we marked all passages in the literature naming or giving information about antecedents and outcomes. Under antecedents, we understood those variables that have been shown to be preconditions for a successful (or unsuccessful) HAIT. We included those variables that were discussed or investigated by the respective authors as preceding or being needed for teaming (experience), without having a pre-defined model of antecedents and outcomes in mind. For the outcomes, we summarized the variables that have been found to be affected by the implementation of HAIT in terms of the human and technical part, team and task level, performance, and context. We only looked at those variables that were under examination empirically or centrally discussed within the non-empirical publications. Antecedents or outcomes only named in the introductions or theoretical background were not included, as those did not appear vital within the literature. We synthesized the insights for all clusters and gave an overview over all antecedents and outcomes, quantifying their appearance. This was done by listing each publication's individual variables and then subsequently grouping and sorting the variables within our researcher team to achieve a differentiated, yet abstracted picture about all factors under examination within the field of HAIT.

## 4. Results

### 4.1. Literature network on human-AI teaming

After removing isolated nodes (*n* = 63) without connections and two articles with missing reference meta-data, the network consisted of 299 nodes (i.e., papers) and 2,607 edges (i.e., paths between the publications). Each paper had on average 17.44 edges connected to it. This is in line with the expected network structure, given that a well-defined and curated part of the literature was analyzed, where most papers share references with other papers. The strength (corrected mean strength = 18.23) was slightly higher than the average degree (17.44), showing a small increase in information gained by using a weighted network instead of an unweighted one. The uncorrected mean strength was 200.55. Transitivity, also known as global clustering coefficient, measures the tendency of nodes to cluster together and can range between the 0 and 1, with larger numbers indicating greater interconnectedness (Ebadi and Schiffauerova, 2015). The observed transitivity was 0.36, which is much higher than random degree of clustering, compared to a transitivity of 0.06 of a random graph with the same number of edges and nodes. The network diameter (longest path between two nodes) was 6, and the density (number of possible vs. observed edges) was 0.06. Overall, this shows that the papers analyzed are part of a connected network that also displays clustering, providing further insights about the network's character.

**Figure 3:**
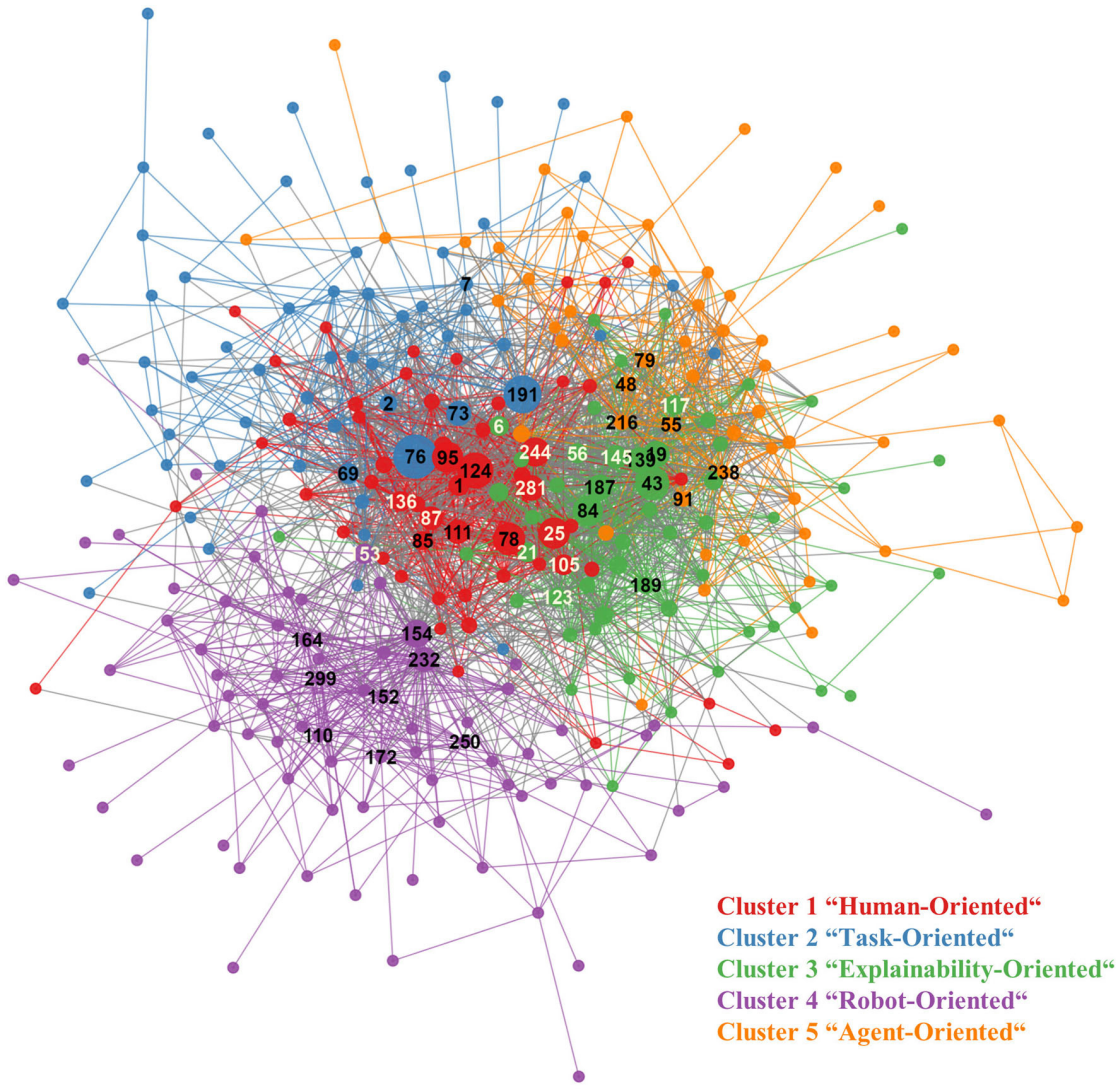
Graph of the bibliometric network. Numbers indicate publications included in the content analysis. Publications are matched to their reference numbers in Table 2. White numbers represent papers included based on their relevance for the whole network, black numbers represent papers selected based on their relevance in their cluster. The clusters' titles will be further explained in section 4.2.

In total, multilevel community clustering identified five clusters that fit our criteria of a cluster size of ≥20 edges (see Figure 3). The sizes for the five clusters were: *n*_1_ = 55, *n*_2_ = 58, *n*_3_ = 55, *n*_4_ = 75, *n*_5_ = 54. Thus, all except two edges could be grouped in these clusters. The modularity of the found cluster solution was 0.36. Modularity is a measure introduced by Newman and Girvan (2004) that describes the quality of a clustering solution A modularity of 0 indicates no better clustering solution than random, while the maximum value of 1 indicates a very strong clustering solution. Our observed modularity of 0.36 fell in the lower range of commonly observed modularity measures of 0.3–0.7 (Newman and Girvan, 2004).

### 4.2. Authors and publication organs within the network

Overall, the network involved about 1,400 authors (including the editors of conference proceedings and anthologies). While most of them were the authors of one to two publications within the network, some stood out with four or more publications: Jonathan Cagan (five papers), Nathan J. McNeese (eight papers) & Beau G. Schelble (four papers), Andre Ponomarev (four papers), Myrthe L. Tielman (four papers) and Dakuo Wang (four papers; see Table 2).

**Table 2:** Composition of the identified clusters and strengths (str.) of the included paper.

**Cluster 1**			**Cluster 2**			**Cluster 3**			**Cluster 4**			**Cluster 5**			**Network's most connected papers**		
**No**.	**References**	**Str**.	**No**.	**References**	**Str**.	**No**.	**References**	**Str**.	**No**.	**References**	**Str**.	**No**.	**References**	**Str**.	**No**.	**References**	**Str. tot**.
124	Vössing et al. (2022)	435	76	Yam et al. (2023)	263	43	Fan et al. (2022)	475	232	Castro et al. (2021)	473	216	Kraus et al. (2021)	254	25	Cabour et al. (2022)	731
95	Xiong et al. (2023)	414	69	Jain et al. (2021)	261	84	Naiseh et al. (2023)	428	154	Mukherjee et al. (2022)	418	48	Chong et al. (2023)	194	244	Weisz et al. (2021)	697
111	O'Neill et al. (2022)	393	191	Jain et al. (2022)	261	187	Silva et al. (2022)	423	164	Rodrigues et al. (2023)	310	79	Chong et al. (2023)	174	281	Johnson et al. (2021)	640
78	Endsley (2023)	388	7	Chandel and Sharma (2023)	194	19	Lai et al. (2022)	379	299	Galin and Meshcheryakov (2021)	224	91	Kridalukmana et al. (2022)	172	87	Le et al. (2023)	599
85	Hauptman et al. (2023)	370	73	Jiang et al. (2022)	183	139	Rastogi et al. (2022)	377	172	Othman and Yang (2022)	192	55	Demir et al. (2021)	168	145	Chen et al. (2022)	590
1	Saßmannshausen et al. (2021)	348	2	Li et al. (2022)	164	189	Tabrez et al. (2022)	353	152	Semeraro et al. (2022)	191	238	Wang et al. (2021)	167	105	Verhagen et al. (2022)	534
									110	Dahl et al. (2022)	189				153	Tsai et al. (2022)	524
									250	Aliev and Antonelli (2021)	184				136	Arslan et al. (2022)	523
															56	Cabitza et al. (2021)	517
															6	Zhang and Amos (2023)	517
															117	Fogliato et al. (2022)	511
															21	Pynadath et al. (2022)	509
															123	Cruz et al. (2021)	489

str., node strength based on the cluster's subgraph; str. tot., node strength based on the main graph; no., number of publications within cluster (see Figure 4).

Looking at publication organs, we list all journals, conference proceedings or anthologies of the respective 10% most connected publications within and across the clusters in Figure 4 for economic reasons. To give further insights, we classified those publication organs according to their thematic focus based on color coding.

**Figure 4:**
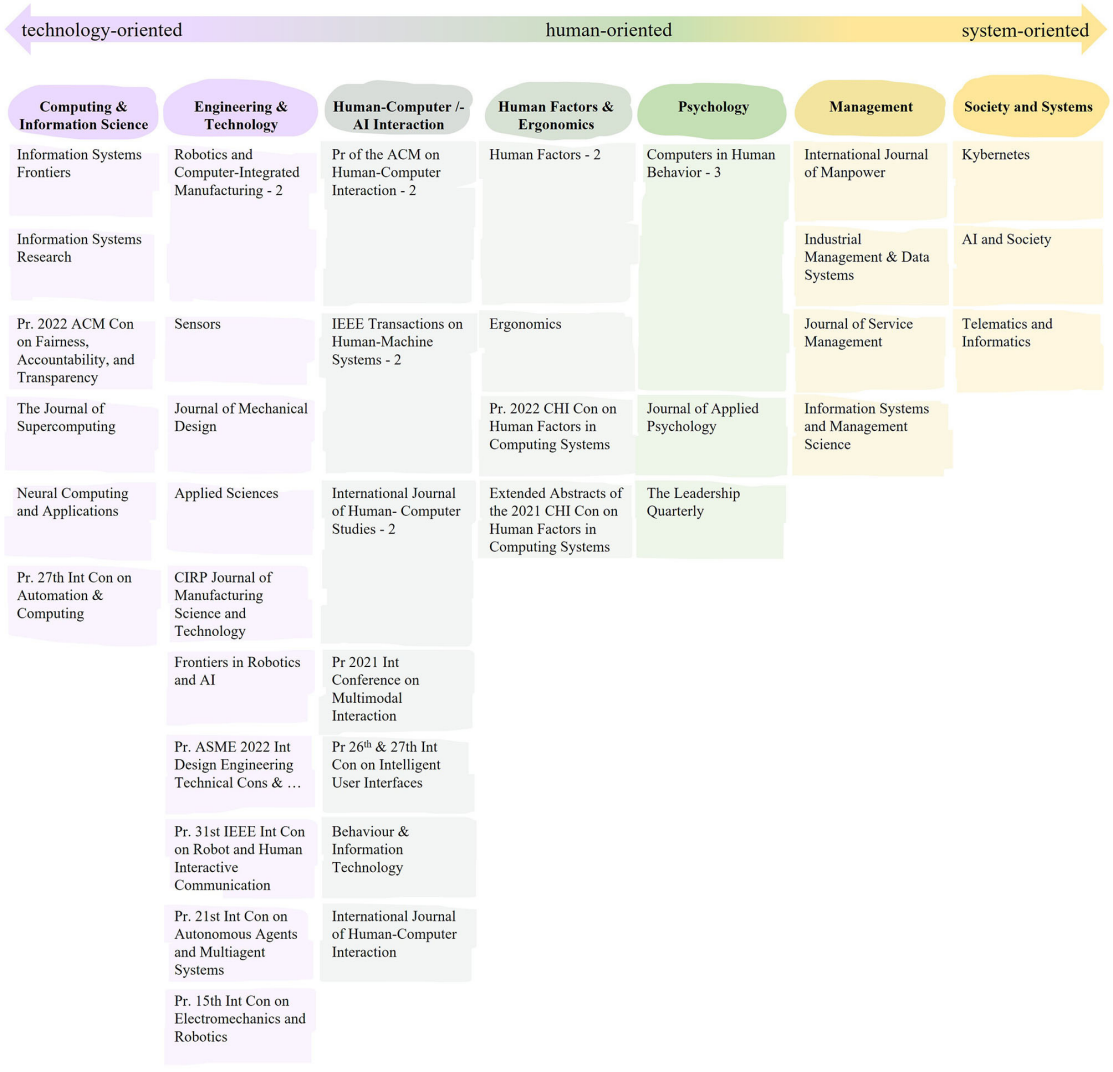
Publication organs within the analyzed papers in the network, sorted by their point of orientation. Pr., proceedings; Con, conference; Int., international.

### 4.3. Description of clusters within the network

For the content analysis, we decided to include the publications with the 10% highest weighted degree from each cluster to deduce the focus in terms of content and research of these identified clusters and in general. Thus, we read six representative contributions for clusters 1, 2, 3, and 5, eight publications from the larger cluster 4, and for the 10% of articles with the highest weighted degree across the network, another 13 publications were screened, resulting in n = 45 publications within the network being reviewed concerning the topic of human-AI teaming.

Regarding RQ1, we subsequently provide a description of the thematic focus within the five clusters. However, it should be acknowledged that the content of theses clusters exhibits a high degree of interconnectedness, making it more challenging to distinguish between them as originally anticipated. The distinctions among the clusters are based on subtle variations in research orientation or the specific AI systems under investigation. A noteworthy commonality across all clusters is the prevailing technical orientation observed in current HAIT research. This orientation is also reflected in the disciplinary backgrounds of the researchers involved, with a predominant presence of computer science and engineering expertise across the clusters and in the whole network and partially in the publication organs. The nuanced aspects of this predominantly one-sided perspective, which we were able to discern, are outlined in the subsequent section. Table 2 provides information on the composition of each cluster, including the contributing researchers and the weighted degree of each contribution.

#### 4.3.1. Cluster 1: human-oriented

The 10% most central articles within this first cluster were all journal articles, mostly from ergonomics and psychology-oriented journals: Three of them belonged to Computers in Human Behavior, while the others were from Human Factors, Ergonomics and Information System Frontiers. Two articles shared the two authors McNeese and Schelble. The papers are not regionally focused, with contributions from the US, Germany, Australia, China and Canada. All take a human-oriented approach to HAIT, looking at or discussing a number of subjective outcomes of HAIT such as human preferences, trust and situation awareness. All the papers seem to follow the goal of finding key influencing factors on the human side for acceptance and willingness to team up with an AI. One exception was the paper by O'Neill et al. (2022), which is based more on the traditional technology-centered LOA model in its argumentation, but still reports on many studies looking at human-centered variables.

#### 4.3.2. Cluster 2: task-oriented AI modes

Whilst the 10% most central articles did not have much in common considering geographic origin, authors, journals and conferences, they share a rooting in information science. All the papers, except for Yam et al. (2023), discuss different types of intelligence automation or roles of AI. They argued from a task perspective, with a focus on the application context and specific ideas for collaboration strategies dependent on the task at hand.

#### 4.3.3. Cluster 3: explainability

The 10% most central articles from Cluster 3 were conference proceedings (four) and journal articles (two) all within the field of human-computer interaction. Three of the articles incorporated practitioner cooperations (with practitioners from Microsoft, Amazon, IBM and/or Twitter). The authors were mainly from the USA, the UK and Canada. Methodologically, the articles were homogeneous in that they all reported laboratory experiments in which a human was tasked with a decision-making scenario during which they were assisted by an AI. The articles took a technical approach to the question of how collaboration, calibrated trust and decision-making can be reached through AI explainability (e.g., local or global explanations, visualizations). Explainability can be defined as an explainer giving a corpus of information to an addressee that enables the latter to understand the system in a certain context (Chazette et al., 2021). The goal of the articles was to facilitate humans to adequately accept or reject AI recommendations based on the explainability of the system. AI has been characterized as an advisor/helper or assistant and the understanding of AI is focused on the *algorithm/machine-in-the-loop paradigm*, involving algorithmic recommendation systems that inform humans in their judgements. This is seen as a fundamental shift from full automation toward collaborative decision-making that supports rather than replaces workers.

#### 4.3.4. Cluster 4: technology-oriented

*Cluster 4* can be described as a *technology-oriented cluster*, which focused primarily on robots as the technology under study. Of the 10% most central articles in this cluster, a majority were journal articles (six), added by two conference contributions. The papers were mainly related to computer science and engineering and similar in their methods, as most of the papers (six) provided literature and theoretical reviews. No similarities could be found regarding the location of publication: While a large part of the articles included in Cluster 4 were published in Europe (Portugal, Scotland, UK, Sweden, and Italy), there were also contributions from Canada, Brazil, and Russia. All included papers dealt with human-robot collaboration as a specific, embodied form of AI, with an overarching focus on the security aspects during this collaboration. The goal of the incorporated studies was to identify factors that are important for a successful collaboration in a modern human-robot collaboration. In this context, communication emerged as an important influencing component, taking place also on a physical level in the case of embodied agents, which necessitates special consideration of security aspects. Furthermore, the articles had a rather technology-oriented approach to safety aspects in common and in most of the articles, concrete suggestions for the development and application of robot perception systems were made. Nevertheless, the papers also discussed the importance of taking human aspects into account in this specific form of collaboration. Additionally, they shared a common understanding of the robot as a collaborative team partner whose cooperation with humans goes beyond simple interaction.

#### 4.3.5. Cluster 5: agent-oriented

The 10% most connected articles within the cluster consisted of conference proceedings (five) and one journal article, all from the fields of human-machine systems and engineering. The authors were mostly from the USA, but also from Germany, Australia, Japan, China and Indonesia and from the field of technology/engineering or psychology. Methodologically, the papers all reported on laboratory or online experiments/simulations. A connecting element between the articles was the exploration of how human trust and confidence in AI is formed based on AI performance/failure. One exception is the paper by Wang et al. (2021), which is a panel invitation on the topic of designing human-AI collaboration. Although it announced a discussion on a broader set of design issues for effective human-AI collaboration, it also addressed the question of AI failure and human trust in AI. In general, the articles postulated that with increasing intelligence, autonomous machines will become teammates rather than tools and should thus be seen as collaboration partners and social actors in human-AI collaborative tasks. The goal of the articles was to investigate how the technical accuracy of AI affects human perceptions of AI and performance outcomes.

The main focus of the clusters, similarities as well as differences are summarized in Table 3. Taken together, the description of the individual clusters reveals slightly different streams of current research on HAIT and related constructs, within the scope of more technology-driven research yet interested in the interaction with humans.

**Table 3:** Description of the clusters.

	**Cluster 1**	**Cluster 2**	**Cluster 3**	**Cluster 4**	**Cluster 5**
**Perspective**	**Human-oriented**	**Task-oriented**	**Explainability-oriented**	**Robot-oriented**	**Agent-oriented**
Methods	Mainly mixed methods, qualitative interviews, field and online experiments, literature review	Mixed methods, vignette study, theory and framework development, literature synthetization, commentary, experiment, experience sampling	Mixed-methods, laboratory experiments with Wizard of OZ or real AI	Mainly theory and framework development, literature review, partly enriched with exemplary studies	Laboratory and online experiments, panel invitation
Forms of AI	Decision (support) system, variety of software or embodied agents	Decision (support) system, robot	Decision (support) system, virtual drone	Robots with machine or reinforcement learning techniques	Embodied agents, software
Role and understanding of AI	Different roles from decision support to mutually supporting team member, augmentor of intelligence, support in decision-making, full, active member with an own role, equal partner, social counterpart	Different roles, augmentor of intelligence, decision agent, independent, active agent, partner & teammate	Assistant & helper, advisor	Autonomous agent, (physical) interaction partner	Autonomous agent, conversational agent, partner, teammate rather than tool
Terms for HAIT	Cooperation, human-AI collaboration, human-autonomy-teaming, human-machine team, human-AI teaming	Human-AI cooperation, collaboration, augmented intelligence, human-computer symbiosis	Human-AI collaboration, collaborative partnership, algorithm-in-the-loop, AI-assisted decision-making, human-AI partnership, human-agent/robot/drone teaming	Human-robot interaction, human-robot collaboration, duality, human-robot team	Mixed-initiative interactions, human-AI collaboration, human-AI teaming, autonomy as teammate
Understanding of HAIT	Independent agents working toward a common goal, adaptive roles within team	Differentiated understanding from independent to interdependent, integrated architecture	Spectrum from full automation to full human agency, AI assistance to support humans	Supportive relationship, working together for task accomplishment, co-working with influence of each's values and broadening individual capabilities	Complementary strengths, prompt interaction in response to communication flow, adaptation toward dynamic task to achieve common goal
Contexts under examination	Hospitality, production management, cyber incident response, sequential risky, decision-making, context-free	Context-aware services, managerial decision-making, financial markets, gig economy platforms, autonomous driving	Clinical decision making, user experience, content moderation, performance prediction, gaming	Manufacturing, production, industry, safety, context-free	Design, military, autonomous driving, context-free

### 4.4. Understandings of human-AI teaming

To answer RQ2 on understandings of human-AI teaming and to find patterns in terminology and definitions potentially relevant for the research question on a common ground definition, the following section deals with the understandings of human-AI teams that emerged from the individual clusters and the overarching 10% highly weighted papers.

Within *cluster 1*, there were several definitions and defining phrases in the papers. The most prominent and elaborate within the cluster might be that of O'Neill et al. (2022), underlining that “If [the AI systems] are not recognized by humans as team members, there is no HAT” (p. 907) and defining human-autonomy teaming as “interdependence in activity and outcomes involving one or more humans and one or more autonomous agents, wherein each human and autonomous agent is recognized as a unique team member occupying a distinct role on the team, and in which the members strive to achieve a common goal as a collective” (p. 911). This definition is also referred to by McNeese et al. (2021). To this, the latter added the aspects of dynamic adaptation and changing task responsibility. Endsley (2023) differentiated two different views on human-AI work: one being a supportive AI enhancing human performance (which is more of where Saßmannshausen et al., 2021 and Vössing et al., 2022 position themselves), and one being human-autonomy teams with mutual support and adaptivity (thereby referring to the National Academies of Sciences, Engineering, and Medicine, 2021). What unites those papers' definitions of HAIT are the interdependency, the autonomy of the AI, a shared goal, and dynamic adaptation.

In *cluster 2*, there were not many explicit definitions of HAIT, but a number of terms used to describe it, with “teaming” not being of vital relevance. Overall, the understanding of HAIT—or cooperation—is very differentiated in this cluster, with multiple papers acknowledging that “various modes of cooperation between humans and AI emerge” (Li et al., 2022, p. 1), comparable to when humans cooperate. The focus in these papers lies on acknowledging and describing those differences. Jain et al. (2022) pointed out that there can be different configurations in the division of labor, dependent on work design, “with differences in the nature of interdependence being parallel or sequential, along with or without the presence of specialization” (p. 1). Li et al. (2022) differentiated between inter- and independent behaviors based on cooperation theory (Deutsch, 1949), describing how the preference for those can be dependent on the task goal. Having this differentiation in mind, intelligence augmentation could happen in different modes or by different strategies, as well as mutually, with AI augmenting human or humans augmenting AI (Jain et al., 2021). This led to different roles evolving for humans and robots, although the distinct, active role of AI was underlined as a prerequisite for teaming (Li et al., 2022; Chandel and Sharma, 2023). The authors claimed that research is needed on the different cooperation modes.

In *cluster 3*, the central papers argued that the pursuit of complete AI automation is changing toward the goal of no longer aspiring to replace domain workers, but that AI “should be used to support” their decisions and tasks (Fan et al., 2022, p. 4) by leveraging existing explainability approaches. In that, the aspiration to reach *collaborative processes* between humans and AI was understood as a “step back” from full automation, which becomes necessary due to ethical, legal or safety reasons (e.g., Lai et al., 2022). Collaboration, along with explainability, is a central topic in cluster 3, which Naiseh et al. (2023, p. 1) broadly defined as “human decision-makers and [...] AI system working together”. The goal of human-AI collaboration was defined as “‘complementary performance' (i.e., human + AI > AI and human + AI > human)” (Lai et al., 2022, p. 3), which should be reached by explainability or “algorithm-in-the-loop” designs, i.e., a paradigm in which “AI performs an assistive role by providing prediction or recommendation, while the human decision maker makes the final call” (Lai et al., 2022, p. 3). Thus, the understanding of human-AI teaming was based on the perspective that AI should serve humans as an “assistant” (Fan et al., 2022; Lai et al., 2022; Tabrez et al., 2022) or “helper” (Rastogi et al., 2022); the notion of AI being a “team member” was only used peripherally in the cluster and HAIT was not explicitly defined as a central concept by the selected papers of cluster 3.

In *cluster 4*, which focused mainly on robots as technological implementations of AI, the term teaming was not used once to describe the way humans and AI (or humans and robots) work together. The terms “human-robot interaction” (HRI) and “human-robot collaboration” (HRC) were used much more frequently, with a similar understanding throughout the cluster: An interaction was described as “any kind of action that involves another human being or robot” (Castro et al., 2021, p. 5), where the actual “connection [of both parties] is limited” (Othman and Yang, 2022, p. 1). Collaboration, instead, was understood as “a human and a robot becom[ing] partners [and] reinforcing [each other]” (Galin and Meshcheryakov, 2021, p. 176) in accomplishing work and working toward a shared goal (Mukherjee et al., 2022). Thus, the understanding of collaboration in cluster 4 is similar to the understanding in Cluster 3, differentiating between distinct roles in collaboration as in Cluster 2. The roles that were distinguished in this cluster are the human as a (a) supervisor, (b) subordinate part or (c) peer of the robot (Othman and Yang, 2022). A unique property of cluster 4 involved collaboration that could occur through explicit physical contact or also in a contactless, information-based manner (Mukherjee et al., 2022). The authors shared the understanding that “collaboration [is] one particular case of interaction” (Castro et al., 2021, p. 5; Othman and Yang, 2022) and that this type of interaction will become even more relevant in the future, aiming to “perceive the [technology] as a full-fledged partner” (Galin and Meshcheryakov, 2021, p. 183). However, more research on human-related variables would be needed to implement this in what has been largely a technology-dominated research area (Semeraro et al., 2022).

In *cluster 5*, the understanding of HAIT is based on the central argument that advancing technology means that AI is no longer just a “tool” but, due to anthropomorphic design and intelligent functions, becomes an “effective and empowering” team member (Chong et al., 2023, p. 2) and thus a “social actor” (Kraus et al., 2021, p. 131). The understanding of AI as a team member was only critically reflected in the invitation to the panel discussion by Wang et al. (2021) who mentioned potential “pseudo-collaboration” and raised the question of whether the view of AI as a team member is actually the most helpful perspective for designing AI systems. The shift from automation to autonomy has been stressed as a prerequisite for effective teaming. Thus, rather than understanding HAIT as a step back from full automation (see cluster 3), incorporating autonomous agents as teammates into collaborative decision-making tasks was seen as the desirable end goal that becomes realistic due to technological progress.

**Figure 5:**
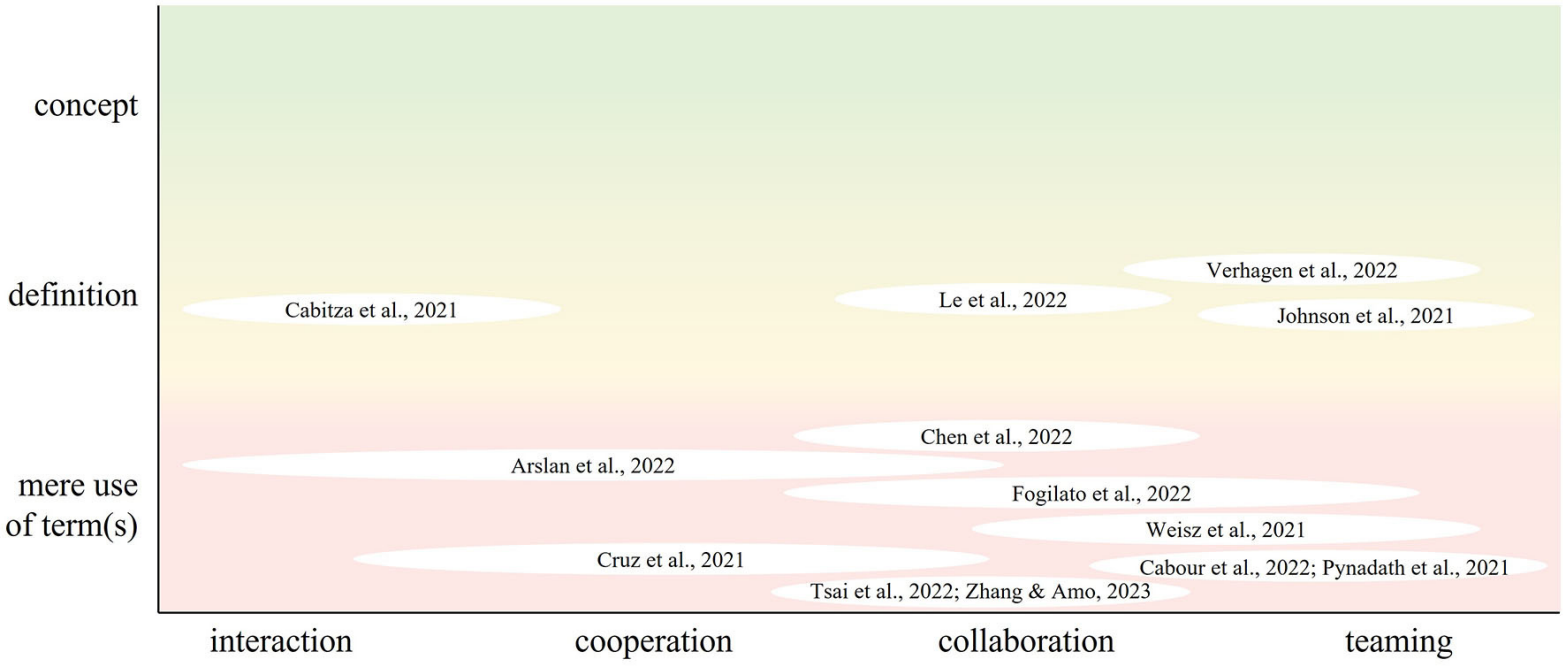
Papers with the most impactful connections within the network on HAIT, classified according to their definitory approach and their use of terms for teaming. “Mere use of term(s)” refers to using one of the listed terms without employing or referencing a definition. “Definition” includes the articles in which the understanding of the used teaming term is specified. “Concept” refers to a deep understanding toward the used term, e.g., by differentiating it from other terms or deriving/proposing a definition.

In addition to the clusters and their interpretation of teaming, we looked at the *10% papers with the highest weighted degree in the whole network*, i.e., the papers that had the most central reference lists across all the literature on HAIT. We expected those papers to deliver some “common sense” about the core topic of our research, as they are central within the network and connected with papers from all clusters. Contrary to our expectations, none of those articles focused on trying to classify and differentiate the concept of HAIT from other existing terminologies in order to create a common understanding across disciplines. See Figure 5 for a classification of the articles based on the extent to which the construct was defined in relation to the term used to depict collaboration.

Four of the central papers showed attempts to define HAIT or related constructs: In the context of human-robot teaming, Verhagen et al. (2022) explored the concept of HART (human-agent/robot team), which encompassed the collaboration and coordination between humans and robots in joint activities, either acting independently or in a synchronized manner. A key aspect emphasized by the authors is the need for mutual trust and understanding within human-robot teams. Similarly, the study conducted by Le et al. (2023) also used robots as interaction partners, although the terminology used was “collaboration”. They drew a comparison between the streams of research focusing on human-robot collaboration, which is technically oriented, and human-human collaboration, which is design oriented. To develop their approach to human-robot collaboration, they considered not only the relevant literature on collaboration, but also the theory of interdependence (Thibaut and Kelley, 1959). In turn, Johnson et al. (2021) discussed the concept of human-autonomy teaming and emphasized the importance of communication, coordination, and trust at the team level, similar to Verhagen et al. (2022). Their perspective was consistent with the traditional understanding of teaming, recognizing these elements as critical factors for successful teamwork. Another perspective was taken by Cabitza et al. (2021) who used the term “interaction” to a large extent including AI not only for dyadic interaction with humans but also as a supportive tool for human decision teams. They emphasized a contrast to the conventional understanding of human-AI interaction, which views AI either as a tool or as an autonomous agent capable of replacing humans (Cabitza et al., 2021).

The remaining papers referred to HAIT or related constructs in their work but provided minimal to no definition or references for their understanding: Arslan et al. (2022) emphasized that AI technologies are evolving “beyond their role as just tool[s]” (Arslan et al., 2022, p. 77) and are becoming visible players in their own right. They primarily used the term “interaction” and occasionally “collaboration”, focusing on the team level without delving into the characteristics and processes of actual teaming. Cabour et al. (2022), similar to Cruz et al. (2021), discussed HAIT only within the context of explainable AI, without providing a detailed definition or explanation. Cruz et al. (2021) specifically used the term “human-robot interaction” rather than teaming, where the robot provides explanations of its actions to a human who is not directly involved in the task. Emphasizing the “dynamic experience” (Chen et al., 2022, p. 549) of both parties adapting to each other, Chen et al. (2022) used mostly the term “human-AI collaboration”. They adopted a human-centered perspective on AI and the development of collaboration. In addition, the paper by Tsai et al. (2022) discussed human-robot work, primarily using the notion of collaboration to explore different roles that robots can take, including follower, partner, or leader. The paper by Zhang and Amos (2023) focused on collaboration between humans and algorithms. Fogliato et al. (2022) focused on “AI-assisted decision-making” (p. 1362) and used mainly the term “collaboration” to describe the form of interaction. They only used the term “team” to describe the joint performance output without further elaboration on its characteristics or processes. Weisz et al. (2021) took the notion of teaming a step further, discussing future potential of generative AI as a collaborative partner or teammate for human software engineers. They used terms such as “partnership,” “team,” and “collaboration” to describe the collaborative nature of AI working alongside human engineers. Finally, Pynadath et al. (2022) discussed human-robot teams and emphasized the “synergistic relationship” (p. 749) between robots and humans. However, they also did not provide additional explanations or background information on their understanding of teamwork.

What we see overall is that there are different streams of current research on HAIT, examining different aspects or contexts of HAIT. Whilst there is one cluster centered around human perception of HAIT, with a tendency to use the term teaming, the other clusters focus more on the AI technology or on the task, describing teaming in a sense of cooperation or collaboration, partially envisioning the AI as a supportive element. Also, within the network's most connected papers, we find this diversity in understandings and terminology and, yet again, a lack in conceptual approaches and definitions.

### 4.5. Antecedents and outcomes

To structure the antecedents and outcomes under examination within the clusters on RQ3, we developed a structural framework helping to group them according to the part of the (work) system they refer to. We used the structuring of Saßmannshausen et al. (2021) as an orientation, who differentiate AI characteristics, human characteristics and (decision) situation characteristics as categories for antecedents. As our reference was HAIT and not only the technology part (as with Saßmannshausen et al., 2021), we needed to broaden this scheme and chose the categories of human, AI, team, task (and performance for outcomes) and context to describe the whole sociotechnical system. We as well-added a perception category for each category to clearly distinguish between objectively given inputs (see also O'Neill et al., 2022) and their subjective experience, both being potential (and independent) influence factors or outcomes of HAIT. Note that all antecedents and outcomes were classified as such by the authors of the respective publications (e.g., by stating that “X is needed to form a successful team”) and can relate to either building a team, being successful as a team, creating a feeling of team cohesion etc. The concrete point of reference differs depending on the publication's focus but is always related to teaming of human and AI.

Cluster 1 contained a high number of antecedents of HAIT or variables necessary to it such as trust. Amongst these were the (dynamic) autonomy of the AI, trust, but also aspects relating to explainability of the AI and situation awareness. Two of the papers took a more systematic view on antecedents, structuring them into categories. The review by O'Neill et al. (2022) contained in this cluster, sorts the antecedents they found into characteristics of the autonomous agents, team composition, task characteristics, individual human variables and training. Communication was found to serve as a mediator. Saßmannshausen et al. (2021) structure their researched antecedents (of trust in the AI team partner) into AI characteristics, human characteristics and decision situation characteristics. For outcomes, cluster 1 included—next to a number of performance- and behavioral outcomes—many different subjective outcomes, e.g., perceptions of the AI characteristics, perceived decision authority, mental workload or willingness to collaborate. O'Neill et al. (2022) did not provide empirical data on outcomes of HAIT itself, but presented an overview of the literature on various outcomes, including performance on the individual and team level (70 studies), workload (39 studies), trust (24 studies), situation awareness (23 studies), team coordination (15 studies) and shared mental models (six studies).

Cluster 2 incorporated relatively few antecedents and outcomes of teaming, as most papers focused on the structure or mode of teaming itself. These cooperation modes could be considered as the central antecedent of the cluster. AI design, explainability as well as the specificity of the occupation, task (and goal) or the organizational context were also named. They were supposed to affect subjective variables such as trust, role clarity, attitude toward cooperation and preference for a feedback style, but also broad organizational aspects such as competitive advantages.

In cluster 3, AI explainability emerged as the main antecedent considered by all central articles. The articles differed in the way that explainability was technologically implemented (e.g., local vs. global explanations), but all considered it as an antecedent for explaining outcomes related to calibrated decision-making (objective, i.e., accuracy of decisions as well as subjective, i.e., confidence/trust in decision).

The majority of the contributions in cluster 4 consisted of theoretical reviews and frameworks, in which antecedents of a successful human-robot collaboration were derived and discussed. Identified antecedents, primarily related to the physical component of a robotic system, were robot speed, end-effector force/torque, and operational safety aspects. Indicated antecedents, which were discussed and can also be applied to non-embodied AI systems, were the ability of the system to learn and thus to generalize knowledge and apply it to new situations, as well as effective communication between the cooperation partners, a shared mental model to be able to work toward the same goal, and (bidirectional) trust. In addition, the usability of the system, its adaptability, and ease of programming, the consideration of the psychophysiological state of the human (e.g., fatigue, stress) and the existing roles in the workplace were identified as prerequisites for a create harmonious collaboration between humans and technologies. When considering the antecedents addressed, expected outcomes included increased productivity and efficiency in the workplace, reduced costs, and better data management.

The articles in cluster 5 considered or experimentally manipulated AI performance (accuracy, failure, changes in performance) and the general behavior of the system (proactive dialogue). The articles argued that this is a central antecedent for explaining how trust is developed, lost or calibrated in human-AI teams.

Overall, the antecedents and outcomes on HAIT have received a large amount of research interest, thus a number of variables have already been studied in this context (see Tables 4, 5 for an overview).

**Table 4:** Antecedents of human-AI teaming.

**Category**	**Antecedent**	**No of sources**	**Sources**	**Cluster(s)**
Human	Individual human variables	3	O'Neill et al., 2022; Othman and Yang, 2022; Xiong et al., 2023	1, 4
	Digital affinity, including Aversion to AI	2	Saßmannshausen et al., 2021; Jain et al., 2022	1, 2
	Psychophysiological state	2	Galin and Meshcheryakov, 2021; Mukherjee et al., 2022	4
	Control	1	Vössing et al., 2022	1
	Mental model of situation	1	Endsley, 2023	1
Perception of human	/	0		
AI	Explainability of AI, including Local and global explanations Visualizations/guidance Explanation and information about decision uncertainty Transparency	10	Fan et al., 2022; Kridalukmana et al., 2022; Lai et al., 2022; O'Neill et al., 2022; Rastogi et al., 2022; Tabrez et al., 2022; Vössing et al., 2022; Chandel and Sharma, 2023; Endsley, 2023; Naiseh et al., 2023	1, 2, 3, 5
	Design Minding human cognitive skills and limitations Organization-specific adaptation	3	Jain et al., 2021; Jiang et al., 2022; Vössing et al., 2022	1, 2
	Difficulty of programming	1	Galin and Meshcheryakov, 2021	4
	LOA/AI autonomy Partial vs. full Restrictions in autonomy Proactivity of AI	4	Kraus et al., 2021; Mukherjee et al., 2022; O'Neill et al., 2022; Hauptman et al., 2023	1, 4, 5
	Dynamics AI adaptivity AI adaptability	3	Galin and Meshcheryakov, 2021; Mukherjee et al., 2022; Hauptman et al., 2023	1, 4
	AI performance, including Good vs. bad performance Failures Changes in performance Reliability	3	Demir et al., 2021; O'Neill et al., 2022; Chong et al., 2023	1, 5
	Guaranteed safety of the AI	1	Galin and Meshcheryakov, 2021	4
	Openness to human scrutiny	1	Chandel and Sharma, 2023	2
	Conformability of the AI	1	Galin and Meshcheryakov, 2021	4
Perception of AI	Predictability of AI actions	3	Aliev and Antonelli, 2021; Mukherjee et al., 2022; Hauptman et al., 2023	1, 4
	Perceived AI comprehensibility	1	Saßmannshausen et al., 2021	1
	(Bidirectional) trust, including trusting behavior	4	Saßmannshausen et al., 2021; Mukherjee et al., 2022; Semeraro et al., 2022; Vössing et al., 2022	1, 4
	Perceived AI ability	1	Saßmannshausen et al., 2021	1
Team	Team interaction, including Communication	5	Castro et al., 2021; Demir et al., 2021; Mukherjee et al., 2022; O'Neill et al., 2022; Othman and Yang, 2022	1, 4, 5
	Interdependence between human and AI	1	Li et al., 2022	2
	Human-robot roles	1	Othman and Yang, 2022	4
	Collaboration mode Sequential or parallel task, with or without specialization, AI or human first	1	Jain et al., 2022	2
	Team composition (members)	1	O'Neill et al., 2022	1
	Team experience level	1	Hauptman et al., 2023	1
	Shared mental models		Castro et al., 2021; Mukherjee et al., 2022	4
	Situation awareness (SA) Shared SA Human SA of AI state AI SA on state of human	1	Endsley, 2023	1
Perception of team	/	0		
Task	Task characteristics	2	Mukherjee et al., 2022; O'Neill et al., 2022	1, 4
	Work phase	1	Hauptman et al., 2023	1
	Goal orientation (task)	1	Li et al., 2022	2
	Time for decision making	1	Rastogi et al., 2022	3
Perception of task	Ease of critical information transferring	1	Othman and Yang, 2022	4
Context	Effects of the (joint) decision Probability of significant and irreversible changes	1	Hauptman et al., 2023	1
	Training/learning, including Time needed or used for acceptance and understanding of AI	3	Castro et al., 2021; O'Neill et al., 2022; Hauptman et al., 2023	1, 4
	Type of workspace	1	Mukherjee et al., 2022	4

**Table 5:** Outcomes of human-AI teaming.

**Category**	**Outcome**	**No of sources**	**Sources**	**Cluster(s)**
Human	Human agency	2	Fan et al., 2022; Tabrez et al., 2022	3
	Preference for feedback	1	Jain et al., 2022	2
Perception of human	Perceived decision authority	1	Xiong et al., 2023	1
	Subjective workload	2	Lai et al., 2022; Xiong et al., 2023	1, 3
	Fatigue	2	Galin and Meshcheryakov, 2021; Semeraro et al., 2022	4
	Stress	2	Galin and Meshcheryakov, 2021; Tabrez et al., 2022	3, 4
	Fear	1	Galin and Meshcheryakov, 2021	4
	Role clarity	1	Jain et al., 2022	2
AI	/	0		
Perception of AI	Trust/confidence in AI	12	Demir et al., 2021; Kraus et al., 2021; Fan et al., 2022; Jain et al., 2022; Kridalukmana et al., 2022; Rastogi et al., 2022; Tabrez et al., 2022; Vössing et al., 2022; Chong et al., 2023; Endsley, 2023; Naiseh et al., 2023; Xiong et al., 2023	1, 2, 3, 5
	Comfort with AI teammate	1	Hauptman et al., 2023	1
	Acceptance of AI/willingness to collaborate As replacement of a human teampartner	3	Li et al., 2022; Chong et al., 2023; Xiong et al., 2023	1, 2, 5
	AI legitimacy as a team member	1	Hauptman et al., 2023	1
	Social presence	1	Fan et al., 2022	3
	Evaluation of AI autonomy	1	Hauptman et al., 2023	1
	Perceived AI capability/understanding of AI	3	Fan et al., 2022; Lai et al., 2022; Xiong et al., 2023	1, 3
	User experience, including Engagement, subjective perception	3	Fan et al., 2022; Lai et al., 2022; Xiong et al., 2023	1, 3
	Satisfaction with AI	2	Kraus et al., 2021; Fan et al., 2022	3, 5
Team	Human-machine augmentation	1	Chandel and Sharma, 2023	2
	Situation awareness	1	Tabrez et al., 2022	3
	Decision style matching	1	Xiong et al., 2023	1
Perception of team	Interaction experience	1	Galin and Meshcheryakov, 2021	4
	Attitude toward collaboration	1	Li et al., 2022	2
	Preference for a collaboration mode	2	Li et al., 2022; Xiong et al., 2023	1, 2
	Responsibility attribution	1	Xiong et al., 2023	1
Task	Data management	1	Othman and Yang, 2022	4
	Human reliance on AI/adjusted decision making	2	Rastogi et al., 2022; Vössing et al., 2022	1, 3
Perception of task	Perception of task interdependence	1	Xiong et al., 2023	1
Performance	Performance Human performance (e.g., time/number of pauses) AI performance (e.g., efficacy, precision) HAIT performance (e.g., quality of decision)	10	Saßmannshausen et al., 2021; Fan et al., 2022; Lai et al., 2022; Rastogi et al., 2022; Tabrez et al., 2022; Vössing et al., 2022; Chong et al., 2023; Endsley, 2023; Naiseh et al., 2023; Xiong et al., 2023	1, 3, 5
	Cost reduction	1	Othman and Yang, 2022	4
Perceived performance	Perception of efficiency increase through AI	1	Othman and Yang, 2022	4
	Perception of AI performance	1	Xiong et al., 2023	1
	Human confidence in decisions	2	Lai et al., 2022; Tabrez et al., 2022	3
	Confidence in own performance (human)	1	Chong et al., 2023	5
	Perception of task performance	1	Xiong et al., 2023	1
Context	Perceived risk (of a decision)	1	Xiong et al., 2023	1
	Trust in the team by stakeholders	1	Hauptman et al., 2023	1

### 4.6. Definition of human-AI teaming

Our final RQ4 was to identify, if feasible, a cohesive definition that would bridge the diverse aspects addressed in current HAIT research. However, as evident from the results of the other research questions, a lack of defining approaches and concepts is apparent throughout the network. We only found one elaborate definition with O'Neill et al. (2022), which was also cited, but not by the breadth of publications. Notably, the included publications, including O'Neill et al. (2022), predominantly adopt a perspective that focuses on one of the two subsystems within a team (i.e., the human or the AI), and tend to be primarily technology-oriented. That means that it is mainly examined which conditions a technical system needs for teaming or, which characteristics the human being should bring along and how these can be promoted for collaboration. This one-sided inclination is also reflected in the addressed antecedents and outcomes (see Tables 4, 5).

However, in order to foster a seamless teaming experience and promote effective collaboration, it is crucial to consider the team-level perspective as a primary focus. Questions regarding the requisite qualities for optimal human-AI teams and the means to measure or collect these qualities remain largely unaddressed in the included publications, resulting in a blind spot in the network and the current state of HAIT research, despite the fundamental reliance on the concept of teaming. While the review of O'Neill et al. (2022) on human-autonomy teaming dedicates efforts toward defining the concept and offering insights into their understanding, an extension of this concept, particularly with regard to the team-level perspective, is needed. The subsequent sections of the discussion will expound on the reasons for this need in greater detail and propose an integrative definition that endeavors to unite all relevant perspectives.

## 5. Discussion

In this work, we aimed to examine the current scope and breadth of literature of HAIT as well as research streams to comprehend the study field, the existing understandings of the term and important antecedents and outcomes. For this purpose, we conducted a bibliometric network analysis revealing five main clusters, followed by a scoping review examining the content and quality of the research field. Before delving into the terminology and understanding of HAIT and what we can conclude from the antecedents and outcomes under examination, we point out the boundaries and connected risks of our work. This serves as the background for our interpretation and the following idea of conceptualizing and defining the construct of HAIT, which is complemented by demands for future research from a perspective on humane work-design and socio-technics.

### 5.1. Limitations

Choosing our concrete approach of a bibliometric network analysis and follow-up scoping review helped us answer our research questions, despite posing some boundaries on the opportunity of insight. First of all, the chosen methods determined the kind of insights possible. Network analyses rely on citation data to establish connections between publications (Bredahl, 2022). Thereby, the quality and completeness of the citation data may vary, leading to missing or insufficient citations of certain publications, thus causing bias and underrepresentation of certain papers or research directions (Kleminski et al., 2022). We are not aware of a bias toward certain journals, geographic regions or disciplines within our network, but do not know if this also holds for the cited literature. This might lead to certain areas of HAIT research, such as literature on the teaming level, not being considered by the broad body of literature or by the most connected papers (maybe also due to the mentioned inconsistent terminology), which would reflect also in the papers' content revealing blind spots. Furthermore, bibliometric network analyses focus mainly on the structural properties of the network and hence often disregard contextual information (Bornmann and Daniel, 2008), which is why we decided to conduct a scoping review additionally. Scoping reviews are characterized by a broad coverage of the research area (Arksey and O'Malley, 2005), which is both a strength and a weakness of the method: On the one hand, a comprehensive picture of the object of investigation emerges, but on the other hand, a limitation in the depth of detail as well as in the transparency of quality becomes apparent. Only being able to look into the 10% most connected papers within each cluster also limited our opportunity to go into more detail and map the whole field of research, again with the risk of leaving blind spots that are actually covered by literature. Hence, we considered also the most connected papers within the whole network to get a broader picture.

The basis of our network analysis and review was a literature search in WoS and Scopus. Although these are the most comprehensive databases available (Kumpulainen and Seppänen, 2022), there is a possibility that some relevant work are not listed there or were not identified by our search and screening strategy. More than in the databases, this problem might lie in restricting our search to publications published from 2021 onwards. It might be that important conceptual and definitory approaches can be found in the prior years, although we found no indications for that within the qualitative analyses of terminology or referenced definitions. Confining our search strongly to the last 2 years of research enabled us to address a relatively wide spectrum of the latest literature in a field that is very hyped and has a large output of articles and conference contributions. While there is a risk associated with excluding “older” research, we sought to partially balance it out by analyzing the papers' content, including their references to older definitions and concepts. Nonetheless, it remains a concern that our conclusions may primarily apply to the very latest research stream, potentially overlooking an influential stream of, for instance, team-level research on HAIT, that held prominence just a year earlier. Therefore, it is important to view our results as representing the latest research streams in HAIT.

Finally, bibliometric studies analyze only the literature of a given topic and time period (Lima and de Assis Carlos Filho, 2019), which can limit our results because of research not being found under the selected search terms, and the clustering algorithms used are based on partially random processes (Yang et al., 2016), which limits transparency on how results are achieved. We tried to balance this out by properly documenting our whole analysis procedure and all decisions taken within the analysis.

Another limitation was discovered in our results during the analyses. Our primary idea was to find different clusters in the body of literature which illuminate the construct HAIT from different disciplinary perspectives. From this, we wanted to extract the, potentially discipline-specific, understandings of HAIT and compare them among the clusters. Although we identified five clusters approaching HAIT with different research foci, they did not differ structurally in their disciplinary orientation. The differences in terminology and understanding within the clusters sometimes were just as high as between. Almost all of the identified publications, as well as most of the clusters, took a more technology-centered perspective, which means that some disciplines are not broadly covered in our work. For example, psychological, legal, societal, and ethical perspectives are poorly represented in our literature network. An explanation for this may be that there has been little research on HAIT from these disciplines, or that publications within the network that were not included in the review on a content base or literature form former years not included in our network highlighted these perspectives. Finally, it should be noted that even though very different aspects are researched and focused on within the clusters, the understanding of the construct of HAIT within which the research takes place is either not addressed in detail or only in very specific aspects, limiting our ability to answer our RQ2 adequately.

### 5.2. Looking at the results: what we know about HAIT so far

Summarizing the findings within our literature network on HAIT under examination or discussion, we can identify some general trends, but also some research gaps and contradictions.

#### 5.2.1. Current research streams and understandings

To answer *RQ1* about human-AI teaming research clusters, we identified five distinct clusters with varying emphases. Despite their shared focus on technological design while considering human aspects, which also reflects in the network metrics, subtle differences in research foci and the specific AI systems under investigation were discernible: Cluster 1 focuses mainly on human variables that are important for teaming. Cluster 2 examines task-dependent variables. Cluster 3 especially investigates the explainability of AI systems, cluster 4 concentrates on robotic systems as special AI applications, and cluster 5 deals mainly with the effects of AI performance on humans' perception. Except for cluster 1, the publications exhibit a focus on technology and are grounded in engineering principles. This is reflected in the publication organs, which are mainly technically oriented, with many at the intersection of human and AI, but primarily adopting a technological perspective. While other perspectives exist, they are not as prevalent. While reasonable due to technological system development's origin in this field (Picon, 2004), research should allocate equal or even more attention to the human and team component in in socio-technical systems. Human perceptions can impact performance (Yang and Choi, 2014), contrasting with technological systems that perform independently of perceptions and emotions (Šukjurovs et al., 2019). However, current research streams continue to emphasize the technological aspects.

Regarding *RQ2*, both terminologies and their comprehension within the clusters were examined to investigate the understanding of HAIT. A broad range of terms is used, often inconsistently within publications. While “teaming” is occasionally used, broader terms like “interaction” and “cooperation” prevail, with “collaboration” being the most common. Interestingly, many terms used do not focus on the relational or interactional part of teaming but instead highlight technology as support, a partner or a teammate, reflecting the technology-centeredness once again. In parallel, it becomes apparent that the phenomena of work between humans and AI systems are rarely defined or classified by the authors. Instead, the terms “cooperation,” “collaboration,” “interaction,” and “teaming” are used in a taken-for-granted and synonymous manner. Paradoxically, a differentiated understanding emerges in some of the papers: “interaction” denotes shared workspace and task execution with sequential order or just any contact between human and AI, “cooperation” involves access to shared resources to gather task-related information, but retains separate work interests, and “collaboration” entails humans and technologies working together on complex, common tasks. However, this differentiation that is very established in human-robot interaction research (see, e.g., Othman and Yang, 2022), is not consistently reflected within the majority of papers within our network. Except for O'Neill et al.'s (2022) paper, the term “teaming” is underdefined or unclassified in other works. Possible reasons include the dominance of a technology-centric perspective (Semeraro et al., 2022) in current research efforts, as collaboration aspects are likely to attract more interest in other research domains, such as psychology or occupational science (Bütepage and Kragic, 2017). Regarding the exemplary publication organs, those are underrepresented in our network. Another possible reason could be the novelty of the research field of teaming with autonomous agents (McNeese et al., 2021). Compared to the other definable constructs, the concept of teaming has only been increasingly used in recent years, which means that research in this field is still in its infancy and, thus, it has not yet fully crystallized what the defining aspects of teaming are. However, it raises questions about conducting high-quality research in the absence of a well-defined construct, as terms like “teammate” or “partner” alone lack the scientific clarity required for construct delineation.

One interesting idea shown in some of the publications offers a way to unite the different terms used within the field: the concept of existing collaboration modes or different views on human-AI work. Authors such as McNeese et al. (2021), Li et al. (2022), Chandel and Sharma (2023), and Endsley (2023) address that there might be different ways (or degrees) of AI and humans collaborating: Some aim to support the human, which reflects more of a cooperative perspective with distinct, not necessarily mutually interdependent tasks. Others are conceptualized as human-AI teams from the very beginning, with mutual intelligence augmentation, dynamic adaptation to one another and collaborative task execution. One can discuss if these should be seen as different categories of interaction, or if they are considered different points on a continuum of working together.

#### 5.2.2. Antecedents and outcomes

To answer *RQ3* on antecedents and outcomes of HAIT we note that for antecedents, nearly all components of a human-AI team were under examination or discussion at least in a few publications, except for team and human perception. Research on AI characteristics dominated the field, with many constructs under research from the, apparently most important, topic of explainability (10 publications) to dynamics and levels of automation of AI. For team variables, most papers looked at team interaction as well as the conglomerate of (shared) situation awareness and mental models. What we can see overall is a focus on characteristics of the work system, but also quite a few perceptional and subjective antecedents under investigation. This shows the importance of considering not only objectively given or changeable characteristics, e.g., in AI design, but also how humans interact with those characteristics, how they perceive them on a cognitive and affective level.

For the outcomes, we find that trust (11 publications) and performance (10 publications) are by far the most researched and discussed outcomes of human-AI teaming. This is interesting, as they represent both the objective, economically important side of implementing teams of AI and humans, but also the subjective basis for efficient long-term collaboration. In the studies, we find a strong focus on subjective outcomes, considering the perception of oneself within the work situation (e.g., stress or fear), the perception of the AI (e.g., comfort with it, perceived capabilities) which is a focus of the literature with 26 mentions, and the perception of the team (e.g., preference for a collaboration mode) as well as its performance.

Nevertheless, considering human perception in researching and designing HAIT is only the first step toward reaching human-centeredness. This approach portrays the human as the central role within complex sociotechnical systems (Huchler, 2015). As a research philosophy, it goes beyond measuring trust or including some worker interviews in one's research and understands the human (and, e.g., their trust in an AI system) as the starting point of any system design. This perspective perfectly goes along with other conceptual approaches such as a socio-technical thinking (see, e.g., Emery, 1993) or the idea of Industry 5.0 (Breque et al., 2021). The breadth of different antecedents and outcomes found in the field of literature on HAIT is impressive, showing knowledge on specific aspects on HAIT and an interest in interdisciplinarity and finding out about different aspects preceding or resulting from HAIT. Still, it lacks a conceptual underpinning that is holistically considering the human as the central figure within a work system.

#### 5.2.3. Exploring existing definitions of HAIT

What we can see considering current understandings of human-AI teams is that many of the publications involved some definitory elements, be it the support aspect, shared mental models, or mutual communication, but all were very focused on those (or other) specific aspects. Nearly no publication clearly defined HAIT in their theoretical background as a basis of their work—most publication use it in a way as if it was self-explanatory. Terms for teaming are used inconsistently and differentiations between them are only addressed in some publications on different cooperation modes. However, the range of terminology, as well as the multitude of disciplines and perspectives contributing to the study of HAIT, permit extensive exploration and the generation of numerous fresh insights. This diversity is appropriate for a field of research that is just evolving. Nonetheless, in order to enhance the clarity and cohesiveness of the literature in this field, there is a pressing need for a unified conceptual framework that allows for transparency (Flake and Fried, 2020) and illuminates how the amalgamation of various attributes can effectively shape humans and AI into a team. We were not able to find such a widely accepted, clear and comprehensive definition of HAIT that would fully answer *RQ4*. This is a problem that links back to the research topic of Human-Centered AI at Work and its aim to find common ground in theories and methods. To better answer *RQ4*, we therefore developed an own definition on HAIT, which is derived in section 5.3.3.

### 5.3. What we need for HAIT: integrated, well-defined teaming approaches

Overall, a great interest in HAIT research can be seen. Studies are being published successively on this topic, being connected through a network of references, and many variables are examined. Some of them are investigated extensively, such as explainability or trust, while there is a variety of variables that is more exploratory examined in single studies. What is lacking, however, is a defined construct that would systematize the understanding toward HAIT and lead to unified and more integrated research. There is little effort in creating a unified definition for the teaming aspect of humans and AI working together; rather, the focus is still primarily on how to prepare the technological counterpart for collaborating. The way toward a common ground is still to be gone, but our review helps identify what is needed next.

The different terms used, lack of definitions and concepts, and various understandings of what constitutes “teaming” and what role(s) the AI might take make it difficult to unify research, build common ground, and advance the field. Hence, we see the need for…

1. addressing HAIT from a socio-technical perspective, thus strengthening the teaming idea and human-centeredness.
2. understanding the AI as a team partner able to take roles adaptively instead of holder of one specific role.
3. a clear definition and a distinct terminology, that is grounded in the work so far and that has the potential to be referred to and used in future research.

#### 5.3.1. The teaming idea within human-AI teams from a socio-technical perspective

What we have seen throughout the review is the vast interest in human-related variables, that show the importance of a human-centered understanding and a consideration of the whole socio-technical system when examining and designing HAIT. Still, this interest does not yet result in taking a human- or even team-oriented perspective. One of the few definitory approaches of O'Neill et al. (2022), focusses on what the AI needs to be and contribute to enable teaming, and not on what this teaming actually is. Thus, research needs to take a holistic approach involving multiple disciplines to investigate and design functioning, accepted and adaptable collaboration between humans and AI. This idea is not new in itself, but follows the concept of socio-technical system design (see, e.g., Emery, 1993), where work systems are seen as consisting of a social and a technical subsystem, connected by organization. Central to that is the approach of *joint optimization*, meaning to design both systems together and constantly adapt them to one another so that both systems yield positive outcomes (Appelbaum, 1997). The epitome of this thinking is the idea of human-AI teaming. It incorporates the idea of humans (social systems) and AI (technical system) working together, creating synergies and jointly forming something that goes beyond their individual capabilities, and thus a new social system. Hence, we want to underline the importance of bringing the teaming idea, and established theories and empirical research from human-human teaming, into the field of research on human-AI or human-autonomy teaming. In most of the literature, terms underlining the collaborative element such as *partner, symbiosis* or *teammate* are used as buzzwords without further explanation or without really understanding humans and AI as a sociotechnical system acting as a team. For a clearly defined field of research, future work should therefore think carefully about which construct (e.g., interaction, teaming) is examined and disclose this understanding to the readers. Furthermore, different constructs should not be used synonymously, as this can lead to a deterioration in the quality of research and confusion.

For us, the term and idea of teaming is still central, being reflected in the vast use of associated terms and the omnipresent idea of a new quality of interaction with a development away from the tool perspective, adaptive behavior, and shared mental models. This evokes the need to unite knowledge on (human) teaming with knowledge on AI and human interaction to go a step further and establish a concept of HAIT that is viable for sustaining research and implementing it into practice.

#### 5.3.2. The “role” of AI within the team

Throughout the papers within our network, we have identified various labels and roles for the AI systems described. While most papers primarily focus on one specific role of AI in their investigations, some, such as Endsley (2023), describe different “concepts of operation” (p. 4) like an AI as an aid to a human supervisor, AI as a collaborator, or AI as an overseer and limiter of human performance. She also mentions roles like “coach, trainer or facilitator” (p. 4). These roles can be described by factors like the nature of the task (e.g., exploration and exploitation, see Li et al., 2022), the level of dependence between AI and human, and specialization (Jain et al., 2022). Jain et al. (2022) distinguish between different “work designs”, systematically describing the division of labor between humans and AI in different categories. Beyond the literature screened for our review, there are other papers addressing the systematics of human-AI interaction, such as Gupta and Woolley (2021). One notable example with comprehensive categorization is Dellermann et al. (2021), who differentiate between aspects defining AI-human and human-AI interactions.

**Figure 6:**
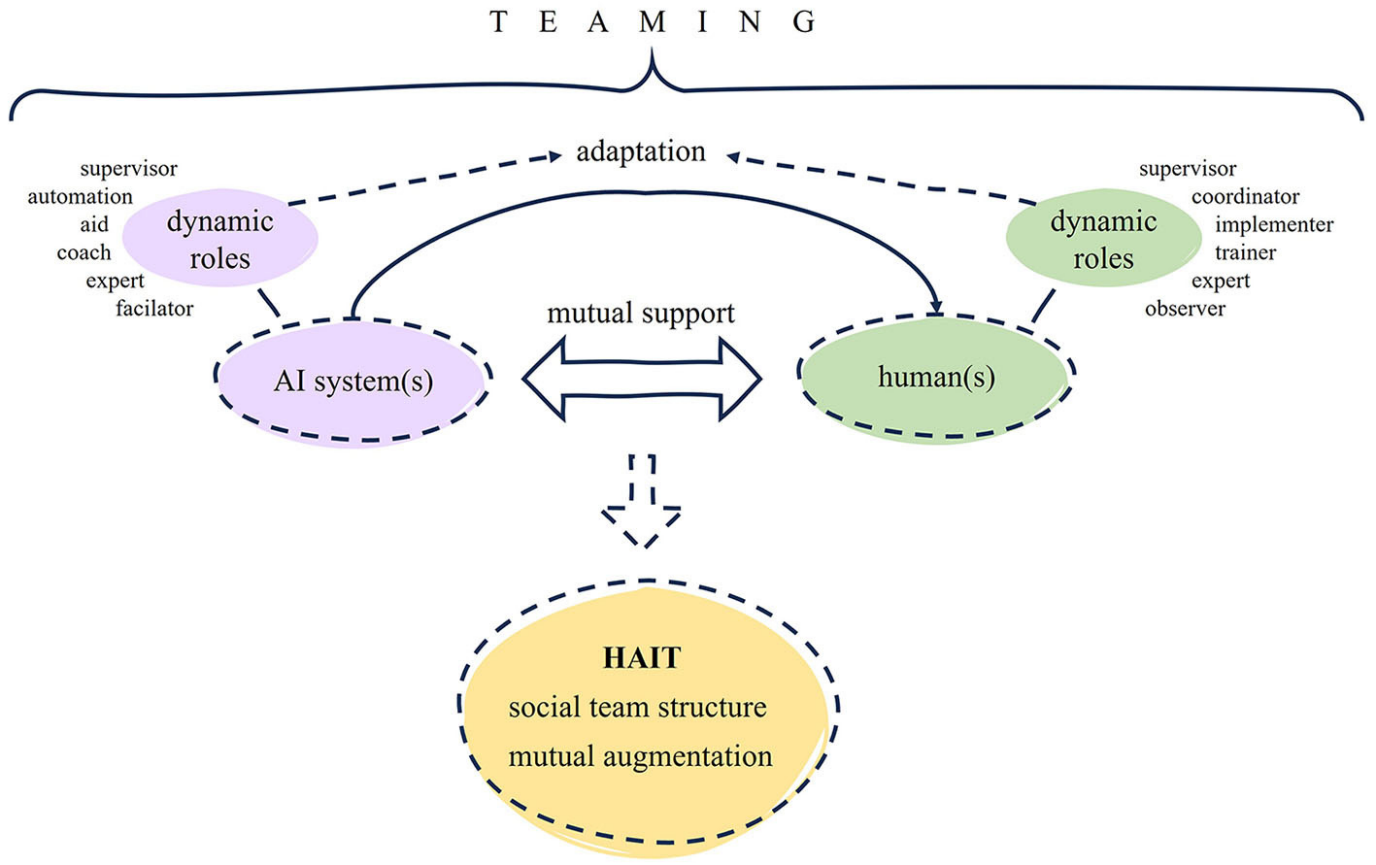
The role of AI within HAIT.

From our perspective, what is needed is to use these existing delineations and taxonomies to develop a new concept of AI as a dynamic team member, capable of adaptively changing roles as required. In our understanding, HAIT goes beyond mere cooperation or collaboration alone, but it can encompass elements of both. HAIT entails humans and AI working together on the same tasks and goals, adapting and exchanging roles as needed. Sometimes, this involves separate cooperation, but it can switch the “mode of collaboration” to mutual support or to the AI providing guidance to the human executor. This understanding of HAIT transcending the categories of cooperation and coordination and including a wide range of potential roles for both humans and AI is depictured in Figure 6.

This concept aligns with the idea of augmented intelligence, as described by Jain et al. (2021), where “computers and humans working together, by design, to enhance one another, such that the intelligence of the resulting system improves” (p. 675). Building on the present research and knowledge about specific roles and cooperation modes, the next step in research is a more realistic, dynamic utilization of AI systems as genuine team members. They should be capable of, e.g., supporting, taking over, cooperating, or setting borders for the human as needed in specific situation. This view of AI as a dynamic team member, akin to humans, can lead to a new, more profound and nuanced understanding of teaming, which now requires a clear definition and appropriate research efforts.

#### 5.3.3. A definition of human-AI teaming

The need for common ground in HAIT research pointed out throughout this paper as well as the whole Frontiers Research Topic “Human-centered AI at work: Common ground and theories and methods”, can, after collating the insights from our review, only be met by a uniting, clear, interdisciplinarity usable definition that is embedded within the idea of socio-technical systems and humane work design. While a diverse research field and evolving insights from different disciplines require the “freedom” to find their own path toward a construct, there comes a point in time where synchronization and integration of perspectives, and necessarily also terminology, become inevitable. This is especially crucial for interdisciplinary exchange, discoverability of publications, discussions employing the same mental models, and transdisciplinary cooperations with practice. Consistent terminology, based on clearly defined and explicit concepts, is a vital prerequisite.

**Figure 7:**
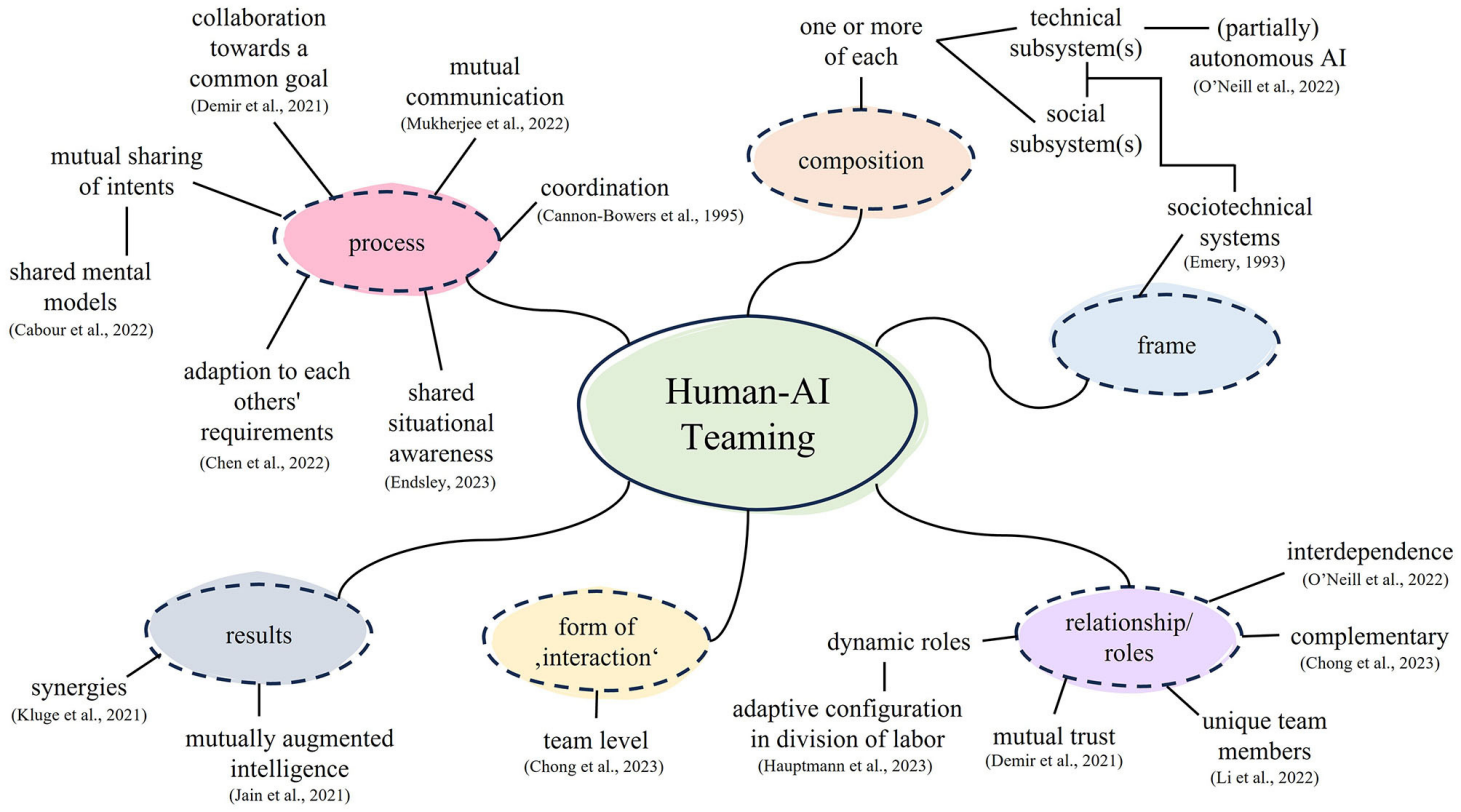
Key components of our proposed HAIT definition. The sources indicated in the figure are exemplary.

After the field of HAIT research has flourished and produced many valuable insights on various various aspects from different disciplines, the time has come for synchronization. As we could not find an appropriate and integrating definition within our literature search, we decided to use the insights from this review, unite them with the theoretical background in human teaming and develop our own definition of HAIT to answer *RQ4*. We base this definition on (1) the theoretical background presented within this paper of human-machine interaction, (2) the theoretical background on human teaming, especially the skill dimensions by Cannon-Bowers et al. (1995), (3) the terms used within the literature on HAIT, and (4) the definitory elements that the different clusters and papers offered. Figure 7 shows an overview of the definitory aspects that we identified throughout this review, together with exemplary sources.

What we propose is a definition of HAIT that is broad enough to unite different research streams yet focuses on the processes and characteristics of teaming rather that that specific to the technology or the human part. This definition enables joint optimization of social and AI-system(s) as they are both equal parts within it and the focal point is the team as a synergetic socio-technical system:

Human-AI teaming is a process between one or more human(s) and one or more (partially) autonomous AI system(s) acting as team members with unique and complementary capabilities, who work interdependently toward a common goal. The team members' roles are dynamically adapting throughout the collaboration, requiring coordination and mutual communication to meet each other's and the task's requirements. For this, a mutual sharing of intents, shared situational awareness and developing shared mental models are necessary, as well as trust within the team.

Our definition centers on the team level, acknowledging its dynamic and changeable nature by understanding HAIT as a process. This emphasis is a response to the prevailing literature on HAIT, which especially highlights the dynamic and adaptive aspects of teaming (e.g., Hauptman et al., 2023). By understanding teaming as a dynamic process, the collaboration system as a whole becomes more flexible compared to narrowly predefined roles and modes of collaboration. This emphasis stems from the recognition of the diverse capabilities and potential applications of AI systems, which have a significant impact on collaboration modes and possibilities. Moreover, the learning ability of AI systems allows their capabilities to evolve and adapt over time (e.g., Mukherjee et al., 2022), further impacting their potential applications. Emphasizing dynamism and adaptivity enables directly addressing of constantly changing contextual and task-related aspects and requirements. Thus, we consider this aspect crucial in our definition, setting it apart from previous definitions, e.g., by O'Neill et al. (2022).

Nevertheless, we do not perceive our definition as a counter-position to O'Neill et al. (2022). On the contrary, all aspects of their definition can be found within ours, making it an extension offering a different focus, namely on the team process, which we identified as a currently blind spot in the literature. Consequently, we have diverged from including specific capabilities of either subsystem in our definition. We have chosen to focus solely on team-level capabilities that contribute to the success of human-AI teams (e.g., shared situational awareness or shared mental models). This choice acknowledges the potential changes in subsystem capabilities resulting from the dynamics and adaptivity of collaboration.

By centering our definition on team processes and capabilities, we hope to offer a useful definition for future research, building upon current research streams on HAIT and considering insights on human teams.

## 6. Key takeaways

**Figure 8:**
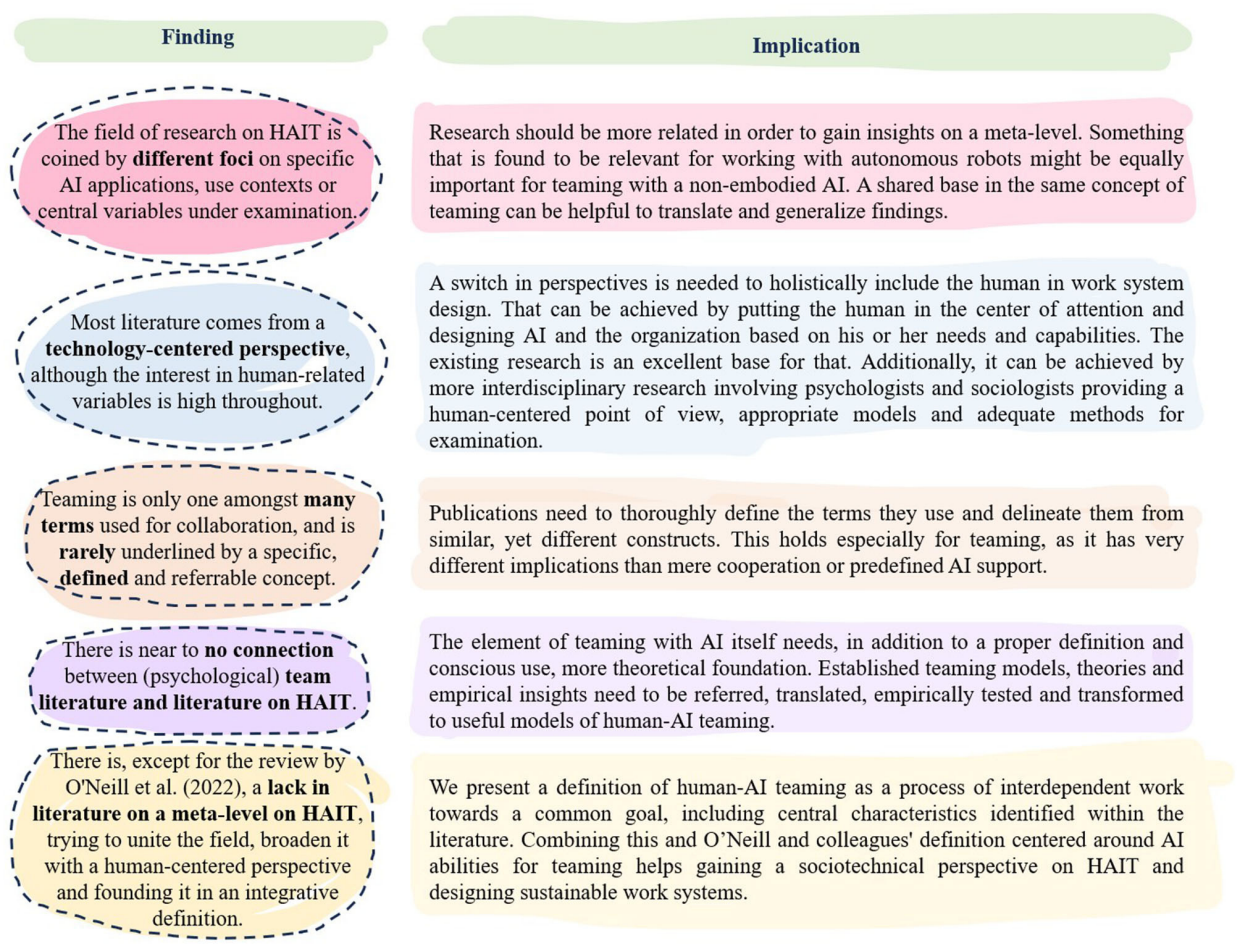
Findings and implications of our work.

Navigating through the field of research, the findings from both our network and content analysis and our interpretation of the results, we want to give the five key findings of the review in Figure 8, each of them leading to a specific practical or theoretical implication.

From a practical point of view, we can conclude that human-AI teaming is still in its infancy. Nevertheless, we see great scientific interest in it as well as many antecedents and outcomes that we already have plenty of knowledge on. Practice, from our point of view, should take inspiration from the fast-evolving research and implement human-AI teaming workplaces. Although this takes much more organizational and work redesign and a more creative and generative approach than just to implement AI as a tool, the opportunities are promising for economic reasons as well as humane work.

## 7. Conclusion

Human-AI teaming is a currently flourishing, multidisciplinary, yet mostly unsystematically approached and so far, one-sided research field. Nevertheless, there is a high need and interest in advancing interdisciplinarity, taking an integrated perspective and finding ways to describe and research a new quality of human collaboration with autonomous technologies, going beyond replacement or mere support of humans in work contexts. Our bibliometric network analysis and scoping review has shown different research streams, understandings, antecedents, and outcomes, revealing the need for a common ground. We close our work by delivering a definition of HAIT considering all the topics from the literature, broadening them with classical teaming knowledge and embedding them in a socio-technical perspective. By this, we want to stimulate future research and promote the convergence of disparate research streams, ultimately fostering the concept of joint optimization in the context of human-AI teams.

## Data availability statement

The raw data supporting the conclusions of this article will be made available by the authors, without undue reservation.

## Author contributions

SB and GO mainly developed the idea of the study. GO took the lead in pre-registration. BG was responsible for the bibliographic network calculation and writing of the respective method sections. AT, GO, and SB were equally responsible for data and content analysis of the articles. SB and AT were in charge of writing the article and of interpreting the results. AK, GO, and BG wrote parts of the paper and, together with CP revised the draft several times to the current state. All authors contributed to the article and approved the submitted version.
