# Supplementary material for: Defining human-AI teaming the human-centered way: a scoping review and network analysis
Source: Front Artif Intell. 2023 Sep 29;6:1250725. doi: 10.3389/frai.2023.1250725 (PMC10570436; doi:10.3389/frai.2023.1250725)
Supplement: Supplementary file 1 [file Table_1.docx]

Supplementary Material

**Defining human-AI teaming the human-centered way: a scoping review and network analysis**

**Sophie Berretta^1†*^, Alina Tausch^1†*^, Greta Ontrup^1^, Björn Gilles^1^, Corinna Peifer^2^, Annette Kluge^1^**

^1^Chair of Work, Organizational, and Business Psychology, Ruhr University Bochum, Germany

^2^Department of Psychology I, University of Lübeck, Germany

^†^These authors contributed equally to this work and share first authorship.

*** Correspondence:**Sophie Berretta, Alina Tausch
[Sophie.berretta@rub.de](mailto:Sophie.berretta@rub.de), [alina.tausch@rub.de](mailto:alina.tausch@rub.de)

# Supplementary Tables

**Table 1**

Skill dimensions for a successful teamwork

| **Skill dimension** | **Definition** |
| --- | --- |
| adaptability | Adaptability is about being able to identify problems and react to them appropriately (Kuehl, 2001). |
| shared situational awareness | Shared situational awareness means understanding each other's activities and sharing perspectives on events and goals. For successful performance, team members need to have a high level of awareness of their specific elements and a similar level of awareness of the shared elements (Gillespie et al., 2013). |
| team management | Team management involves coordinating and monitoring team members' activities, assessing team performance, allocating responsibilities, planning and organizing, as well as creating an atmosphere in which team members can work together (O'Neil et al., 1997). |
| communication | Effective communication is characterized by sharing concise and accurate information with each other (Kuehl, 2001). |
| decision-making | Decision-making refers to the team's ability to make decisions by using and integrating available information to derive logically based judgments, weigh alternatives, assess consequences and select the optimal solution based on these considerations (Kuehl, 2001; O'Neil et al., 1997). |
| coordination | Coordination is defined as the process by which a team organizes resources, activities and responses to achieve integration, synchronization and completion of tasks within specified time boundaries (O'Neil et al., 1997). |
| feedback | Feedback describes the ability of team members to provide, seek and receive advice on how to improve performance in order to contribute to the completion of the task (Salas et al., 2000). |
| interpersonal skills | Interpersonal skills are about improving the quality of interactions and disputes between team members through the use of cooperative behaviors or empowering statements (O'Neil et al., 1997). |

References

Gillespie, B. M., Gwinner, K., Fairweather, N., & Chaboyer, W. (2013). Building shared situational awareness in surgery through distributed dialog. *Journal of Multidisciplinary Healthcare*, *6*, 109–118. https://doi.org/10.2147/JMDH.S40710

Kuehl, M. A. (2001). *Revision of Teamwork Questionnaire for the United States Marine Corps aviation community* [Dissertation]. University of Southern California.

O'Neil, H. F., Allred, K., & Baker, E. L. (1997). Review of Workforce Readiness: Theoretical Frameworks. In H. F. O'Neil (Ed.), *Workforce Readiness: Competencies and Assessment* (pp. 3–25). Psychology Press.

Salas, E., Burke, C. S., & Cannon‐Bowers, J. A. (2000). Teamwork: emerging principles. *International Journal of Management Reviews*, *2*(4), 339–356. https://doi.org/10.1111/1468-2370.00046
